# Single-cell RNA sequencing analysis reveals cell landscape and gene signatures associated with granulomatous lobular mastitis

**DOI:** 10.3389/fimmu.2025.1624640

**Published:** 2025-10-16

**Authors:** Junyue Wang, Yifei Zeng, Mengjie Wang, Dongxiao Zhang, Xulong Zhang, Min Liu, Yudong Li, Xiaolong Xu

**Affiliations:** ^1^ School of Clinical Medicine, Beijing University of Chinese Medicine, Beijing, China; ^2^ Department of Breast, Beijing Hospital of Traditional Chinese Medicine, Capital Medical University, Beijing, China; ^3^ Department of Immunology, School of Basic Medical Sciences, Capital Medical University, Beijing, China; ^4^ Department of Pathology, Beijing Hospital of Traditional Chinese Medicine, Capital Medical University, Beijing, China

**Keywords:** single-cell RNA-seq, granulomatous lobular mastitis, immune system, macrophage, M1 macrophages

## Abstract

**Background:**

Granulomatous lobular mastitis (GLM) is a refractory chronic inflammatory breast disease characterized by granuloma formation and recurrent abscesses, yet its molecular pathogenesis remains poorly understood. To address this knowledge gap, we aimed to systematically compare the immune microenvironment between GLM and healthy breast tissues, reveal disease-associated cellular subpopulations, and characterize key dysregulated genes and pathways driving GLM pathogenesis.

**Methods:**

We performed single-cell RNA sequencing (scRNA-seq) on breast tissue samples from 3 patients with GLM and 3 healthy controls. The sequencing data were subjected to cell clustering, cell abundance comparison, and differential gene analysis to assess immune microenvironment differences. We performed macrophage subtyping and revealed differentially expressed genes. Using GO/KEGG analysis, we characterized signaling pathway disparities in M1 macrophages to investigate potential pathogenic mechanisms.

**Results:**

11 major cell types were detected through scRNA-seq. In GLM tissues, immune cell infiltration was significantly increased (*P* < 0.05), with macrophages and neutrophils showing predominant infiltration. Macrophages were further classified into M1, M2a, M2b, and M2c subtypes, with M1 being the most predominant. In M1 macrophages, we observed marked upregulation of: *FCGR1A* (CD64), *CYBB* and *NCF1* (core NADPH oxidase components), *TNFSF10* (TRAIL). Cytokine signaling and phagocytosis-related pathways were significantly enriched in M1 macrophages.

**Conclusion:**

To our knowledge, this is the first scRNA-seq study of GLM, identifying 11 major cellular populations and implicating macrophages—especially M1 subtype—as central to disease immunopathology. We report dysregulated expression of CD64, NADPH oxidase components, and TRAIL, prompting the hypothesis that phagocytic function may be impaired and nominating this axis as a potential therapeutic target.

## Graphical Abstract

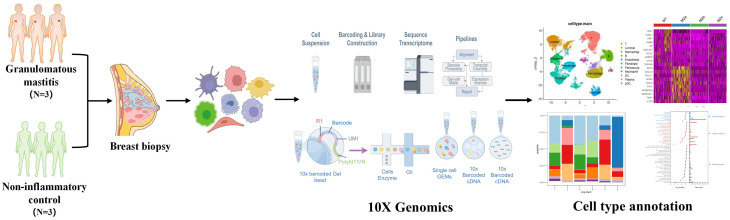

## 1 Introduction

Granulomatous lobular mastitis (GLM) is a chronic, idiopathic inflammatory condition of the mammary gland, characterized by significant pain and a high tendency for recurrence. The incidence of GLM has been gradually increasing, making it a serious breast disease that should not be ignored (1). Pathological examination is typically used to diagnose GLM, which manifests as granulomatous lesions and fused lesions centered in the breast lobules, accompanied by an abundance of lymphocytes, plasma cells, neutrophils, and abscess. Characteristic cells of the granulomas include macrophage-derived epithelioid histiocytes and Langhans giant cells (2).

The pathogenesis of GLM remains poorly defined. Previous studies indicate that factors such as nipple invagination, elevated prolactin levels, and other elements related to ductal secretions are major predisposing factors for GLM (3). Increased permeability of breast ducts allows immunogenic substances, such as retained milk, to enter the lobular mesenchyme, triggering local inflammation. This inflammation induces the infiltration of immunocompetent cells, leading to delayed-type hypersensitivity and granuloma formation (3, 4). Notably, Corynebacterium kroppenstedtii (CK) and C. parakroppenstedtii (CPK) (5) have been identified as key pathogens in GLM (6–8). Nevertheless, the specific mechanisms by which the above factors trigger inflammatory immunity are unknown, resulting in poor empirical management effects. Although corticosteroids provide transient symptomatic relief, their long-term use can cause serious systemic complications, and severe side effects and breast destruction caused by surgery greatly affect the physical and mental health of patients (9). These clinical challenges underscore the urgent need for mechanism-driven therapies targeting core pathogenic pathways.

Conventional investigative approaches relying on marker gene expression and cellular morphology often fall short in capturing subtle yet pathologically significant alterations in cellular behavior and functional states during disease progression. Furthermore, no studies have comprehensively analyzed all cell types and their genetic activity across the entire breast tissue in GLM. This limitation makes it difficult to fully understand the complex pathological mechanisms of GLM during inflammatory and immune processes. In the pursuit of comprehensive insights, single-cell RNA sequencing (scRNA-seq) has emerged as a powerful tool for simultaneously profiling cell subset compositions and cell type–specific transcriptional states (10–12), facilitating the discovery of critical cell subsets and biomarkers that drive pathological changes (13, 14).

Given the limited understanding of immune disorders in GLM and the urgent need to elucidate its pathogenesis, we conducted a comprehensive analysis of GLM and normal breast tissues using scRNA-seq. Our primary objective was to investigate differences in the immune environment of GLM tissues and identify key genes and pathogenic pathways at the cell subset level, offering novel insights into the disease’s pathogenesis.

## 2 Materials and methods

### 2.1 Study design

**Figure 1:**
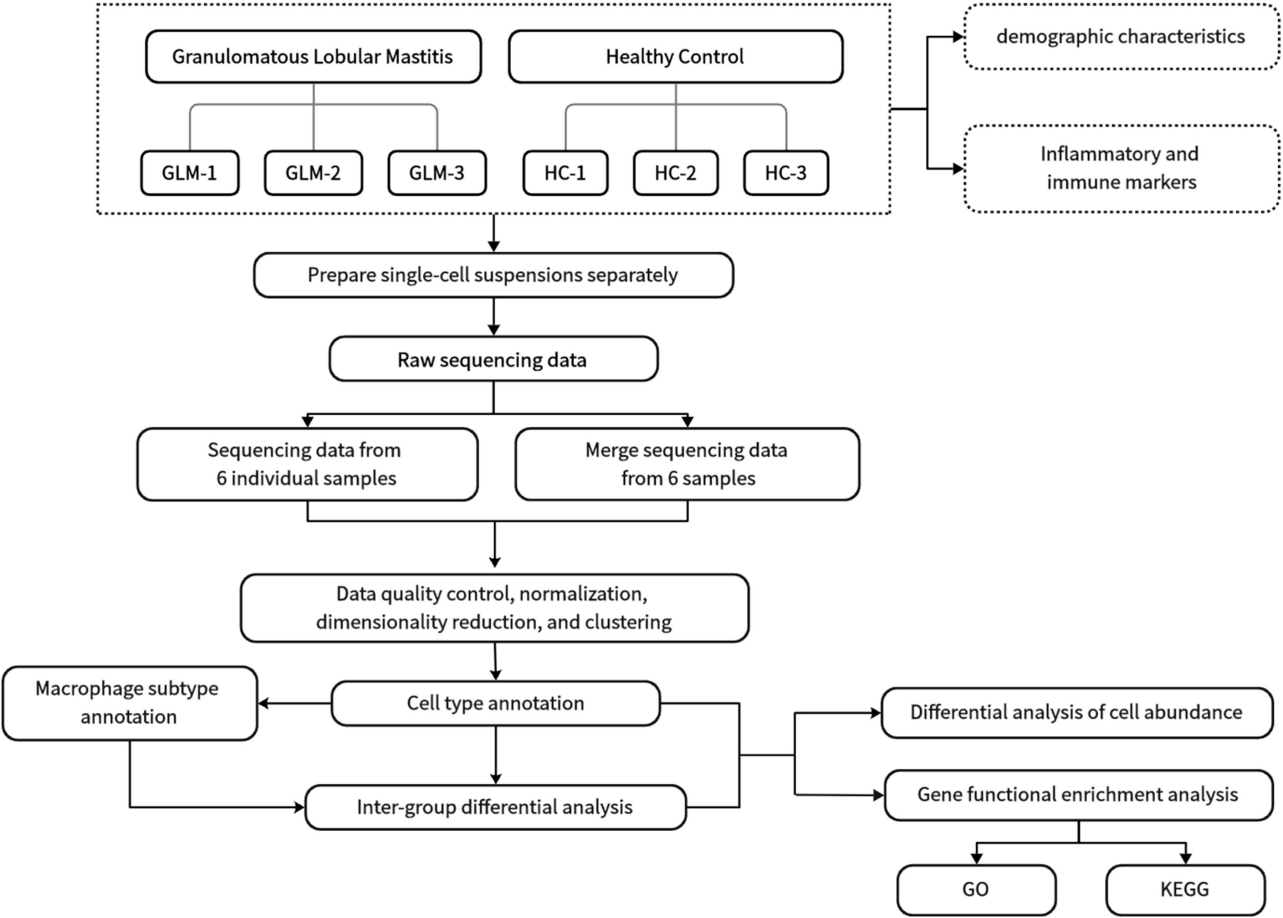
Flow chart of study procedures.

Three GLM patients and three healthy controls (HC) were enrolled in this single cell sequencing study from the Department of Breast at Beijing Hospital of Traditional Chinese Medicine. GLM patients met the diagnostic criteria outlined in the “Management of granulomatous lobular mastitis: an international multidisciplinary consensus (2021).” The research procedure flowchart is presented in **Figure 1**. All patients completed the relevant medical records, including age, lactation history, main symptoms and signs, white blood cell count, and NEUT%. Serum prolactin, erythrocyte sedimentation rate (ESR), CRP, complement C3 (C3), complement C4 (C4), complement C1q (C1q), B-factor (BF), immunoglobulin M (IgM), immunoglobulin A (IgA), immunoglobulin G (IgG), immunoglobulin G4 (IgG4), and immunoglobulin E (IgE) were also measured in the GLM group. Paraffin-embedded tissue sections from GLM patients were stained with hematoxylin and eosin (HE). The single-cell sequencing study was approved by the Ethics Committee of Beijing Hospital of Traditional Chinese Medicine, and all enrolled patients and healthy controls provided informed consent (No.2023BL02-120-02).

### 2.2 Tissue samples from the 3 GLM patients and 3 healthy controls were profiled using the 10X Genomics Chromium Single Cell 3' platform

Fresh tissue samples were surgically obtained. ScRNA-seq was performed using the 10X Chromium microfluidics system (10X Genomic). Tissue samples from the 3 GLM patients and 3 healthy controls were profiled using the 10X Genomics Chromium Single Cell 3' platform. Barcoded cDNA libraries were prepared using the Single Cell 3′mRNA kit. Cell Ranger v3 (3.1.0) was used to demultiplex cellular barcodes and map reads to the human genome (GRCh38-3.0.0) (15).

### 2.3 Processing of single-cell RNA sequencing data

#### 2.3.1 Data integration and quality control

Raw scRNA-seq data were integrated and filtered using the Seurat R package (v3.0.0) (16). Cells were retained based on the following criteria: 1) More than 15% of the transcriptome genes were mapped to mitochondrial genes; 2) the total number of unique transcribed genes in a cell was less than 500; and 3) the total number of unique transcribed genes in a cell was more than 4000. Hemoglobin genes present in scRNA objects were identified based on the following genes: “*HBA1*”, “*HBA2*”, “*HBB*”, “*HBD*”, “*HBE1*”, “*HBG1*”, “*HBG2*”, “*HBM*”, “*HBQ1*”, and “*HBZ*”. The relative expression percentage of these genes in each cell was calculated. Cells meeting these criteria were selected for subsequent analysis.

#### 2.3.2 Normalization and dimensionality reduction

The NormalizeData function was employed to normalize the sequencing data, and 2000 highly variable genes were selected for scaling and principal component analysis (PCA). The Harmony R package (v0.1.0) was utilized to correct for batch effects (17). The appropriate dimensions of Harmony embeddings were then used for downstream uniform manifold approximation and projection (UMAP) visualization and clustering.

#### 2.3.3 Cell type annotation

Cells were broadly classified into three categories: immune cells (*PTPRC*+), epithelial cells (*EPCAM*+), and stromal cells (*MME*+/*PECAM1*+). Subclustering within these categories detected 11 major cell types, including T cells, B cells, macrophages, dendritic cells (DCs), and fibroblasts, among others (**Table 1**).

**Table 1:** Major cell type marker genes.

Cell type	Markers
FIB	DCN, APOD, LUM, COL1A2, COL1A1
END	PECAM1, VWF, ENG, CLDN5, RAMP2, CDH5
LUM	AR, KRT19, KRT18, KRT8
PVC	ACTA2, RGS5, IGFBP5, STEAP4, MYL9
B Cell	CD19, CD79A, CD79B, MS4A1
PLA	IGLC2, IGLC3, IGHA1, IGHA2, IGHM, IGHG1, JCHAIN, IGHG3, MZB1
T Cell	CD7, CD2, CD3G, CD3E, CD3D
NEU	S100A8, S100A9, CSF3R, FCGR3B, AQP9, SMCHD1, CXCL8
MAC	FCGR3A, CD163, SPP1, GPNMB, ACP5, LIPA, C1QA, CD14
pDC	LILRA4, IL3RA, TCL1A, CLEC4C, CLIC3, IRF8
DC	CD83, CD86, CCR7, HLA-DPB1, BIRC3

FIB, fibroblasts; END, endothelial cells; LUM, luminal cells; PVC, perivascular cells; B, B cells; PLA, plasma cells, T, T cells; NEU, neutrophils; MAC, macrophages; pDC, plasmacytoid dendritic cells; DC, Dendritic cells.

In the macrophage subtyping, we used predefined marker genes in combination with multi-marker panels, UMAP clustering, and differential expression analysis to annotate subtypes between the GLM group and healthy control group. The marker genes for M1 macrophages include *FCGR3A, CD80, CD86, CD68, IL1R1, IL1B, TNF, IL6, TLR2*, and *IFNG*, while M2a is characterized by *TGFB1*, M2b by *CD163* and *CD86*, M2c by *CD68* and *CD163*, and M2d by *VEGFA* (**Table 2**). Dot Plot and UMAP plot further illustrate the expression patterns of these markers and the distribution of the cells (**Supplementary Figures S1A, B**).

**Table 2:** Macrophage subtype marker genes.

Subtype	Markers
M1	FCGR3A, CD80, CD86, CD68, IL1R1, IL1B, TNF, IL6, TLR2, IFNG
M2a	TGFB1
M2b	CD163, CD86
M2c	CD68, CD163
M2d	VEGFA

In the annotation of T cell subtypes, we follow the same strategy as for macrophages and use a predefined set of marker genes for determination. Markers included CD8^+^ effector (GZMB, PRF1, NKG7, CCL5), CD8^+^ naive (CCR7, SELL), CD4^+^ memory (S100A4, IL7R, GPR183) and CD4^+^ naive (CCR7, SELL, TCF7, IL7R). When fine-grained signatures were ambiguous, generalized labels CD4^+^ (CD4, IL7R, CCR7, TCF7, LEF1) and CD8^+^ (CD8A, CD8B, GZMB, PRF1, NKG7, CCL5) were retained. Marker panels are listed in **Supplementary Table S1**.

#### 2.3.4 Definition of differential genes

Differentially expressed genes (DEGs) in this single-cell sequencing analysis were identified using the FindMarkers function in the Seurat package, with the default parameter set to test.use=wilcox. Differentially expressed genes were defined by a Log2(Fold Change)>1 and corrected P-value <0.05, and were subsequently ranked according to their log2(fold change) after screening.

#### 2.3.5 Gene function enrichment analysis

Gene Ontology (GO) functional enrichment analysis was primarily conducted using the clusterProfiler R package for the target genes. The Kyoto Encyclopedia of Genes and Genomes (KEGG) serves as a resource database that catalogs biological systems and their associated gene functions. By enriching the differentially expressed genes, we can predict the potential functions and pathways involved in this study. Functional enrichment was conducted using the clusterProfiler R package. We performed over-representation analysis (ORA) on the set of differentially expressed genes (DEGs) identified by Seurat (Wilcoxon rank-sum test with Benjamini–Hochberg correction). GO analysis was performed with enrichGO (ontology = BP), parameters: pAdjustMethod = “BH”, pvalueCutoff = 0.05, qvalueCutoff = 0.05. KEGG pathway analysis used enrichKEGG (organism = “hsa”), with the same multiple-testing adjustment and gene set size parameters. Enriched pathways were considered significant at FDR (q-value) < 0.05.

#### 2.3.6 Intercellular communication network

We used CellChat (R) to infer and compare intercellular communication between the GLM and healthy control groups. Starting from standardized and annotated Seurat objects, we constructed CellChat objects for each group using cell type as the identity label and loaded CellChatDB.human as the prior database. For each group, we applied identifyOverExpressedGenes and identifyOverExpressedInteractions for preprocessing and enrichment screening, then used computeCommunProb to estimate communication probabilities between cell-type pairs and removed interactions with low cell counts. At the pathway level, we further aggregated the network using computeCommunProbPathway and aggregateNet. The two group-level objects were merged and aligned for between-group comparison; differential bar plots were used to quantify interaction number and interaction strength, and circle plots together with differential heatmaps were used to visualize the number and strength of interactions among different cell populations.

## 3 Results

### 3.1 Demographic characteristics

**Figure 2:**
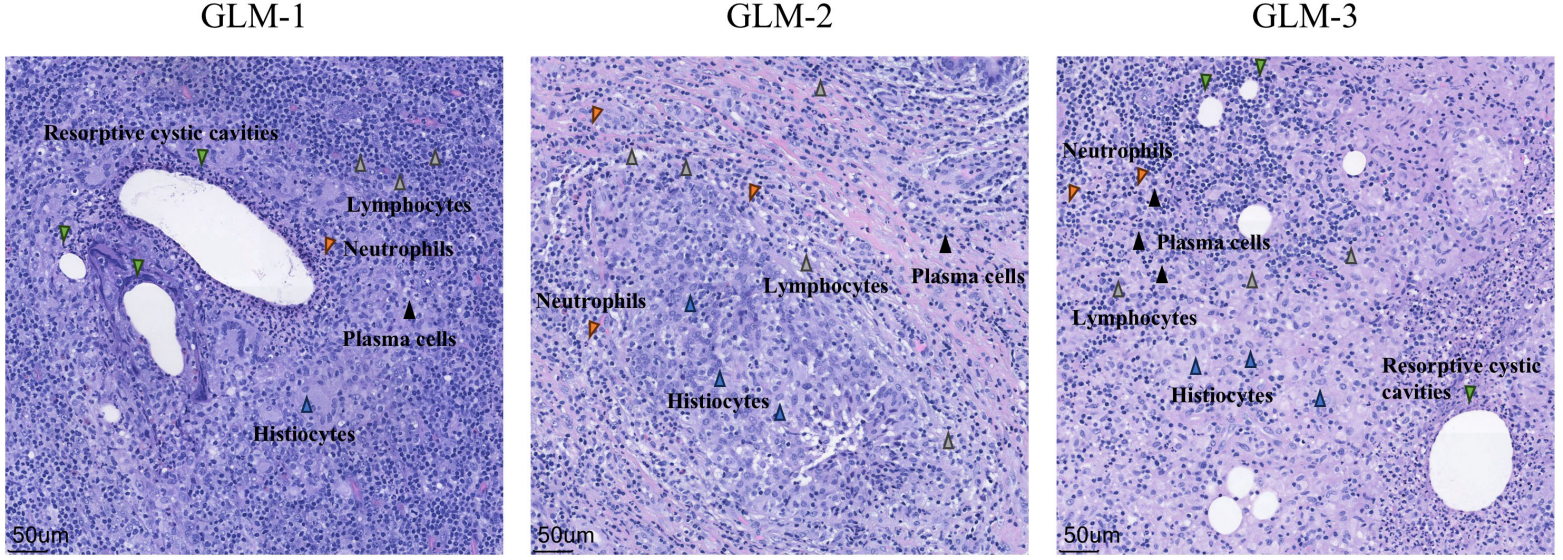
HE staining of pathological tissues from patients with GLM (10X).

Three patients with GLM and three healthy controls were included in this study. There were no significant differences in gender, age, lactation history, WBC and NEUT% between the two groups (P>0.05). This indicates that the baseline characteristics of the two groups were comparable, allowing for subsequent analysis. All patients in the GLM group exhibited clinical manifestations of breast mass, swelling and pain, and increased local skin temperature. Among them, two patients had breast abscesses, and one patient had erythema and arthralgia of the lower limbs. The healthy control group did not show any of these symptoms. Significant differences were observed between the two groups in the clinical manifestations of breast mass, swelling and pain, and increased local skin temperature (P = 0.014) (**Table 3**). In the GLM group, serum prolactin, ESR, CRP, C3, C4, C1q, BF, IgM, IgA, IgG, IgG4, and IgE levels were measured. Among them, ESR, CRP, C3 and C1q were significantly elevated (**Table 4**). Hematoxylin and eosin (HE) staining was performed on pathological breast tissue samples from three patients with GLM. The affected mammary tissue exhibited features of chronic active inflammation, characterized by extensive infiltration of neutrophils, histiocytes, multinucleated giant cells, lymphocytes, and plasma cells. The lobular architecture of the breast was largely obscured. Numerous clusters of histiocytes of varying sizes were observed, with some granulomas merging into confluent sheets. Microabscess formation was noted in the centers of some granulomas, accompanied by resorptive cystic cavities (**Figure 2**).

**Table 3:** Clinical characteristics of patients with GLM patients and health controls.

Demographic Characteristics	GLM (n=3)	HC (n=3)	*P* value
Sex (%)	Female (100%)	Female (100%)	n.s.
Age (years) (mean ± SD)	33.00 ± 3.46	33.33 ± 11.72	0.965
Lactation history (months) (mean ± SD)	15.33 ± 8.50	2.22 ± 3.29	0.067
WBC (10~9/L) [3.50-9.50]	7.68 ± 2.21	5.36 ± 1.09	0.179
NEUT%(%) [40.0-75.0]	70.10 ± 11.02	63.57 ± 7.20	0.438
Breast mass (%)	3 (100%)	0 (0%)	0.014*
Swelling and pain (%)	3 (100%)	0 (0%)	0.014*
High local skin temperature (%)	3 (100%)	0 (0%)	0.014*
Breast abscess (%)	2 (66.67%)	0 (0%)	0.083
Erythema and arthritis (%)	1 (33.33%)	0 (0%)	0.273

n.s.: non-significant; 2) *P < 0.05; 3) WBC, white blood cell; 4) NEUT%, neutrophil percentage.

**Table 4:** Hematological inflammatory and immune-related indicators in GLM patients.

Markers	GLM(n=3)	Normal range	Unit
Serum prolactin	12.08 ± 1.53	2.8 - 29.2	ng\ml
ESR	56.50 ± 57.28 (H)	0 - 20	mm\h
CRP	14.40 ± 19.86 (H)	0.0 - 8.0	mg\L
C3	1.53 ± 0.40 (H)	0.75 - 1.40	g\L
C4	0.30 ± 0.08	0.10 - 0.40	g\L
C1q	284.80 ± 58.60 (H)	159.0 - 233.0	mg\L
BF	348.50 ± 82.59	105.0 - 395.0	mg\L
IgM	1.97 ± 0.15	0.40 - 2.30	g/L
IgA	3.08 ± 1.60	0.70 - 4.00	g/L
IgG	15.48 ± 4.44	7.00 - 16.00	g/L
IgG4	0.50 ± 0.39	0.00 - 2.00	g/L
IgE	58.40 ± 42.56	0.0 - 100.0	IU/mL

H, High.

### 3.2 Identification of major cell types

**Figure 3:**
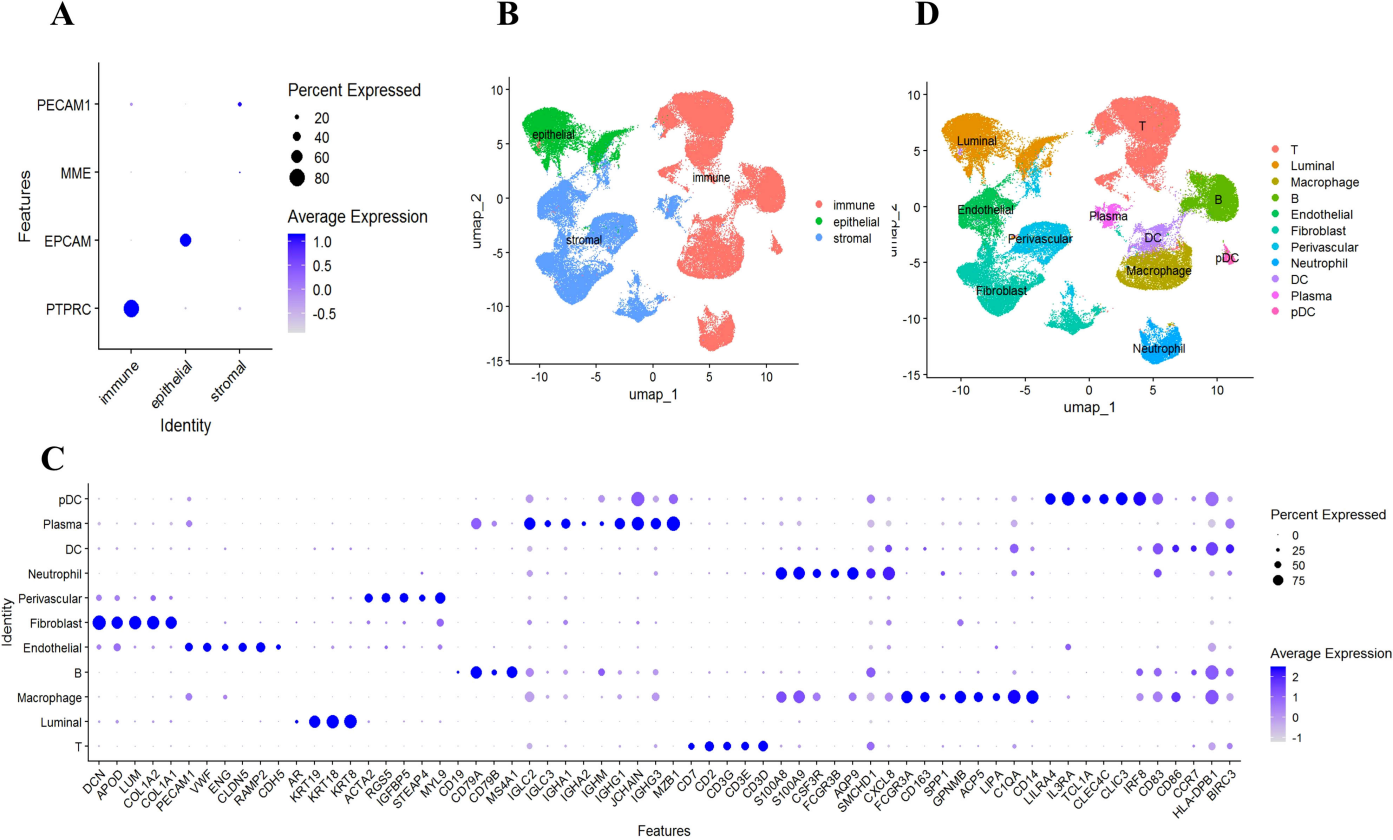
Identification of 11 major cell types. **(A)** Using scRNA-seq data, cells from patients with GLM (n = 3) and healthy controls (n = 3) were labeled as immune cells, epithelial cells, and stromal cells, using marker genes; **(B)** UMAP embeddings depict cells categorized as immune cells, epithelial cells, and stromal cells; **(C)** Marker gene expression for 11 major cell types is illustrated, where dot size and color represent the percentage of marker gene expression (pct. exp) and the averaged scaled expression (avg. exp. scale) value, respectively; **(D)** UMAP embeddings show the 11 major cell types.

To understand the mechanism of GLM at single-cell resolution, we collected tissue samples from six individuals (three GLM patients and three healthy controls) and performed scRNA-seq. We detected 22 subclusters from the scRNA-seq profiles using a UMAP-based clustering method (Methods). Based on the expression of *PTPRC*, *EPCAM*, *MME*, and *PECAM1* (**Figure 3A**), the cell populations were categorized into three groups: immune cells, epithelial cells, and stromal cells (**Figure 3B**). Following cluster analysis, we further annotated 11 major cell types, including T cells, B cells, plasma cells, neutrophils, macrophages, DC cells, pDC cells, endothelial cells, perivascular cells, fibroblasts, and luminal cells. (**Figure 3C**). The distribution of these cell types is shown through UMAP plots (**Figure 3D**). Luminal epithelial cells were the only epithelial subtype robustly identified. We did evaluate canonical myoepithelial markers (*KRT14*, *KRT5*, *TP63*, *MME*) within the epithelial subset; however, these markers showed low/patchy expression and did not yield a stable myoepithelial cluster upon reclustering(**Supplementary Figure S2**).

### 3.3 Intergroup differences in cell population abundance

**Figure 4:**
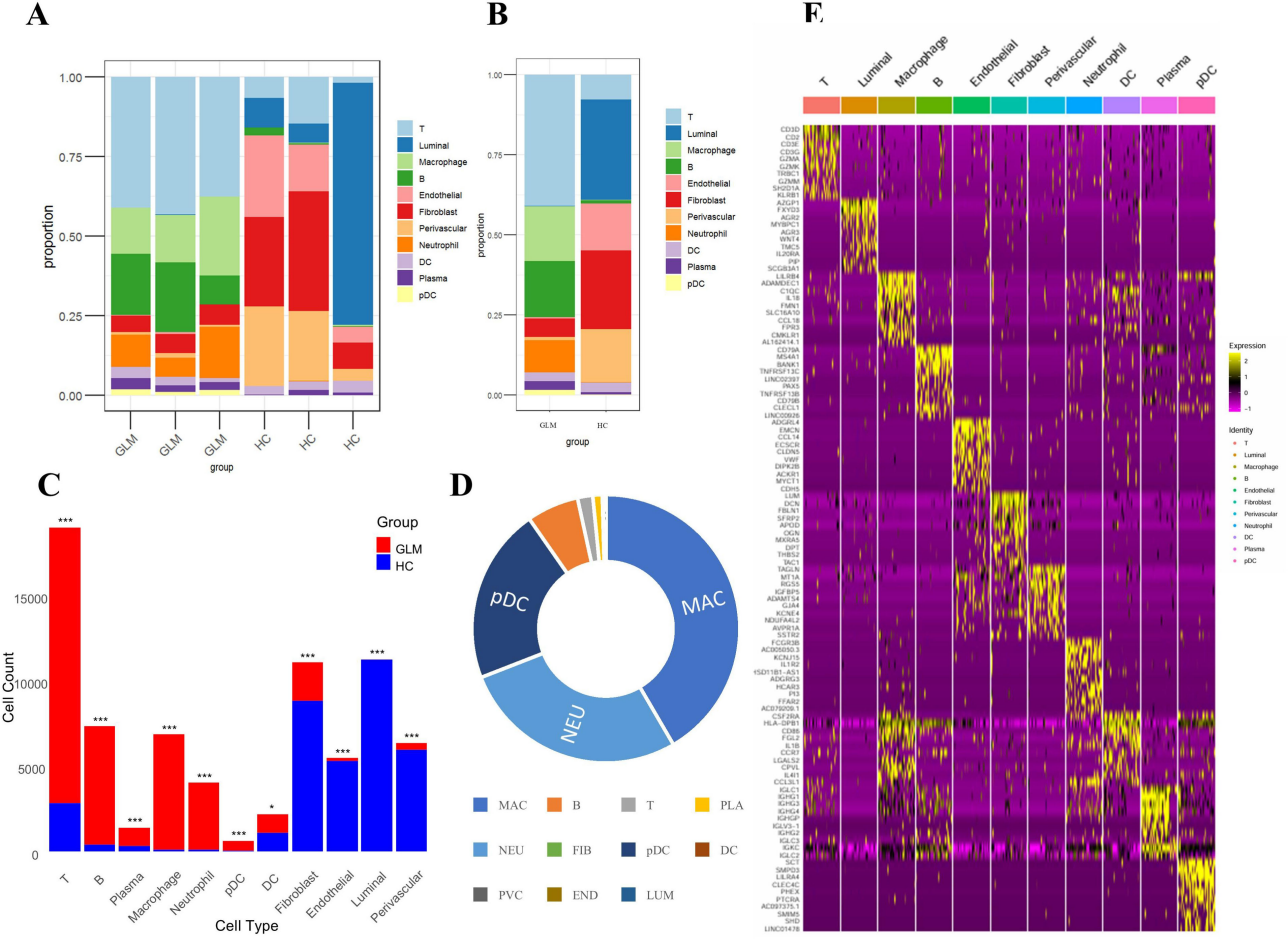
Intergroup differences in cell population abundance and group-specific transcriptional signatures. **(A)** Stacked bar plot showing the proportion of 11 cell populations across samples; **(B)** Comparison of cell population distributions between GLM and healthy controls; **(C)** Statistical analysis of differences in cell abundance between GLM and healthy controls; **(D)** Fold changes between the two groups; **(E)** Heat maps showing cell type–specific gene expression. * indicates a statistically significant difference, *** indicates a difference with high statistical significance.

We compared the abundance of cell populations between the GLM and healthy groups (**Figure 4A**). The three GLM samples exhibited consistent cellular distributions, predominantly composed of immune cells, with significantly higher abundances of macrophages and neutrophils compared to the healthy group. (**Figure 4B**). Similarly, the three healthy samples showed consistent cellular distributions, mainly consisting of fibroblasts and luminal cells (**Figure 4B**). Chi-square analysis revealed statistically significant abundance differences across all 11 cellular populations between GLM patients and healthy controls (*P* < 0.05), with each population exhibiting disease-specific transcriptional alterations (**Figure 4C**). Macrophages and neutrophils showed the largest fold changes between the two groups (**Figure 4D**).

### 3.4 Group-specific transcriptional signatures

We conducted a comprehensive analysis of cell type-specific gene expression patterns in GLM (**Figure 4E**). For example, T cells exhibited high expression of core T cell receptor (TCR) signaling genes (*CD3D*, *CD3E*, *CD3G*) and cytotoxic effector molecules (*GZMA*, *GZMK*, *GZMM*), indicating T cell activation and potential tissue damage mediated by granzymes. Macrophages showed elevated expression of immune-regulatory receptors (*LILRB4*), matrix-degrading enzymes (*ADAMDEC1*), and complement pathway genes (*C1QC*), along with increased levels of inflammatory cytokines (*IL18*) and chemokines (*CCL18*). These findings suggest that macrophages may play a crucial role in the early inflammatory response and in tissue repair and immune regulation, particularly in the context of chronic inflammation or fibrosis. Dendritic cells (DCs) demonstrated high expression of antigen presentation-related genes (*HLA-DPB1*, *CD86*) and chemokines (*CCL3L1*). *HLA-DPB1* is essential for antigen presentation, *CD86* functions as a co-stimulatory molecule for T cell activation, and *CCL3L1* facilitates DC migration and T cell recruitment. These results underscore the role of DCs in bridging T cell recruitment and activation. Plasmacytoid dendritic cells (pDCs) exhibited high expression of inhibitory receptors (*LILRA4*, *CLEC4C)*. Through distinct molecular mechanisms, they regulate pDC activation and functional responses, playing essential roles in modulating immune reactions, preventing excessive immune activation, and maintaining immune homeostasis.

### 3.5 T cell and macrophage heterogeneity

#### 3.5.1 Major T cell subtypes

We subdivided T cells based on the supplementary marker gene sets (**Supplementary Table S1**) using a two-stage decision strategy (first CD4/CD8 lineage, then within each lineage distinguishing naive/memory or effector, with a fallback threshold of 0.05). We ultimately identified CD8^+^ effector T cell, CD8^+^ naive T cell, CD4^+^ memory T cell, CD4^+^ naive T cell, and generalized CD4^+^/CD8^+^ T cell retained when differences were not significant. The distributions of each subtype in the UMAP space are shown in **Supplementary Figure S3**.

#### 3.5.2 Major macrophage subtypes

**Figure 5:**
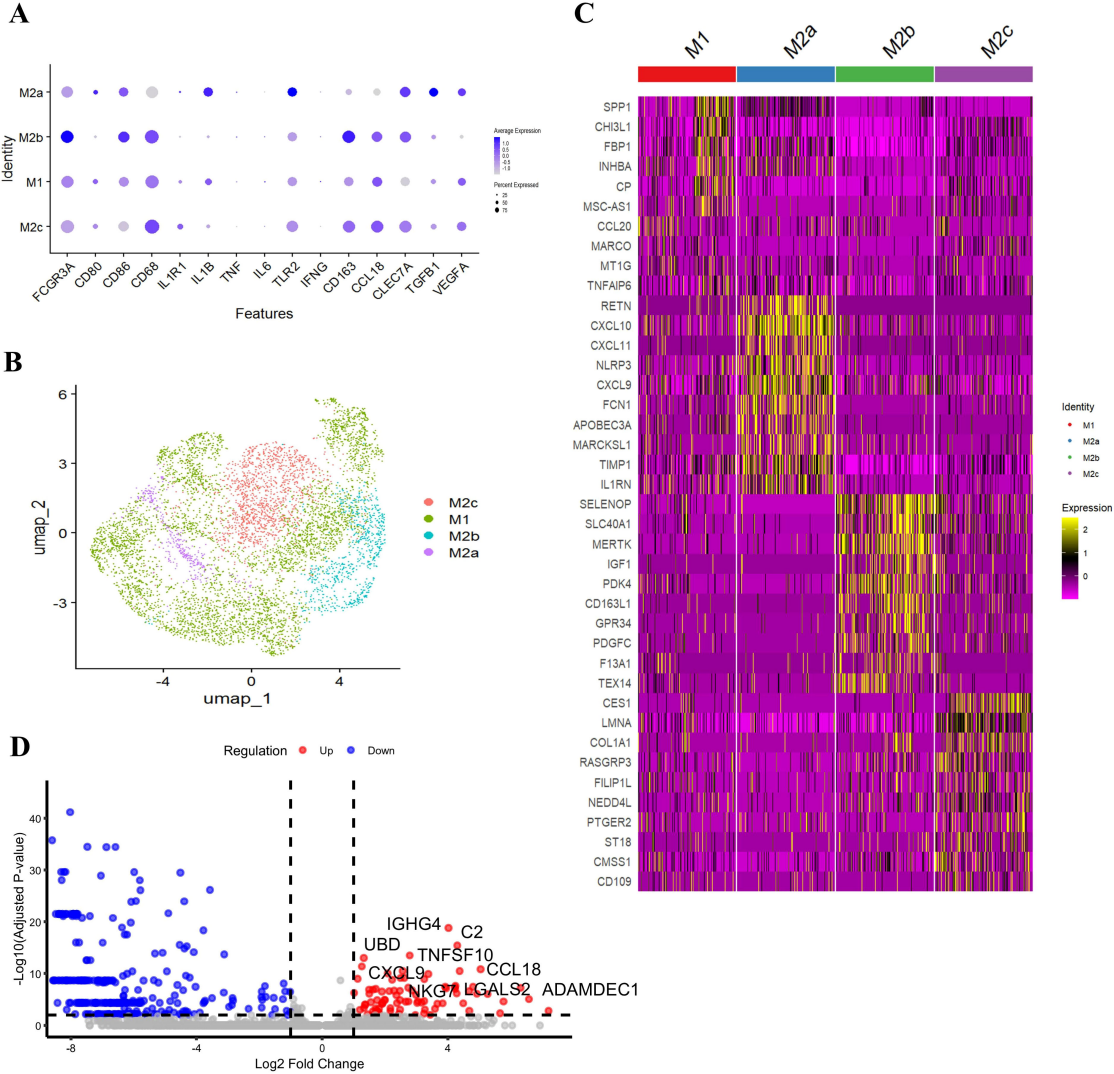
Macrophage subtype heterogeneity and subtype-specific signature genes. **(A)** Identification of Four Macrophage Subtypes in GLM; **(B)** UMAP Plot Showing the Distribution of Macrophage Subtypes; **(C)** Specific Gene Expression of Macrophage Subsets; **(D)** Differentially Expressed Genes Between GLM and HC Groups in M1 Macrophages.

Using the marker genes listed in **Table 2**, we successfully detected four macrophage subtypes—M1, M2a, M2b, and M2c— in GLM tissues (**Figure 5A**). In contrast, M2d macrophages were not prominently detected. The distribution of these macrophage subtypes was visualized using a UMAP plot (**Figure 5B**). Applying the same criteria to healthy tissues, we also detected M1, M2a, M2b, and M2c, but the abundance of M2 subtypes was markedly lower in controls. Because M2a/M2b/M2c counts were very low in healthy samples, statistical power for formal differential testing and inclusion in main figures was limited. To make this explicit, we added side-by-side bar plots and stacked bar plots comparing subtype composition between GLM and healthy groups (**Supplementary Figures S4A, B**). These plots show that M2a/M2b/M2c are present but rare in healthy tissue, whereas they are expanded in GLM, consistent with the inflammatory milieu. However, whether in GLM tissues or healthy breast tissues, M1 macrophages were predominant, accounting for 64.5% and 73.1% respectively.

#### 3.5.3 Functional characterization of macrophage subtypes in GLM

Our study revealed distinct transcriptional features of macrophage subtypes in GLM tissues (**Figure 5C**). M1 macrophages specifically highly expressed *SPP1*, *CCL20*, *TNFAIP6*, etc. *SPP1*, which encodes osteopontin, promotes the recruitment of monocytes/macrophages and the production of cytokines. In certain contexts, *SPP1* ^+^ macrophages exhibit a pro-inflammatory phenotype. *CCL20* is upregulated in various inflammatory diseases and is involved in the recruitment of both pro-inflammatory IL-17-producing helper T cells (Th17) and regulatory T cells (Treg) to sites of inflammation. *TNFAIP6* expression can be induced by pro-inflammatory cytokines such as TNF-α and IL-1.

In contrast, M2a macrophages exhibited high expression of the anti-inflammatory molecule interleukin-1 receptor antagonist (*IL1RN*), alongside upregulation of the *NLRP3* inflammasome. These findings suggest that M2a cells may exert both pro-inflammatory and immunoregulatory functions in the context of chronic inflammation in GLM. Additionally, M2a macrophages showed significant enrichment of Th1-associated chemokines *CXCL9*, *CXCL10*, and *CXCL11*, indicating a potential role in Th1 cell recruitment and the propagation of inflammation. Notably, M2a cells also exhibited elevated expression of resistin (*RETN*) and ficolin-1 (*FCN1*), both of which have been previously associated with chronic inflammation and fibrotic processes (18–20).

Further analysis revealed that M2b macrophages exhibited high expression of *MERTK* and *IGF1*, suggesting potential involvement in anti-inflammatory signaling regulation and cell survival. In contrast, M2c macrophages showed elevated expression of *COL1A1*, indicating a possible role in collagen synthesis and tissue repair.

Subsequently, we conducted an in-depth analysis of differentially expressed genes between the GLM and HC groups in M1 macrophages (**Figure 5D**). In the GLM group, M1 macrophages were characterized by high expression of the complement component *C2*, suggesting that complement-mediated immune activation plays an important role in GLM. Additionally, M1 macrophages were enriched for the Th1-associated chemokine *CXCL9* and the Treg-recruiting chemokine *CCL18*, indicating a potential role in T cell recruitment and shaping of the inflammatory microenvironment. Furthermore, the elevated expression of *TNFSF10* (TRAIL), a TNF-related apoptotic ligand, could affect the activity of NF-κB (nuclear factor-κB) and the expression of its downstream proinflammatory cytokines IL-1β, IL-6, and TNF-α in macrophages (21).

Collectively, our transcriptional profiling allows for a functional characterization of each macrophage subtype in GLM (**Figures 5C, D**). M1 macrophages display a pro-inflammatory signature, marked by genes involved in complement activation (C2) and the recruitment of T cells (CXCL9, CCL18, CCL20). M2a macrophages present a complex phenotype, blending anti-inflammatory potential (IL1RN) and markers linked to fibrosis (RETN, FCN1), suggesting a role in chronic inflammation and tissue remodeling. M2b macrophages, with high expression of MERTK and IGF1, appear poised for immunoregulatory functions and promoting cell survival. Finally, M2c macrophages express COL1A1, indicating a primary role in collagen deposition and tissue repair. This functional heterogeneity underscores the multifaceted contribution of macrophages to GLM pathogenesis.

### 3.6 Functional enrichment analysis of M1 macrophage

**Figure 6:**
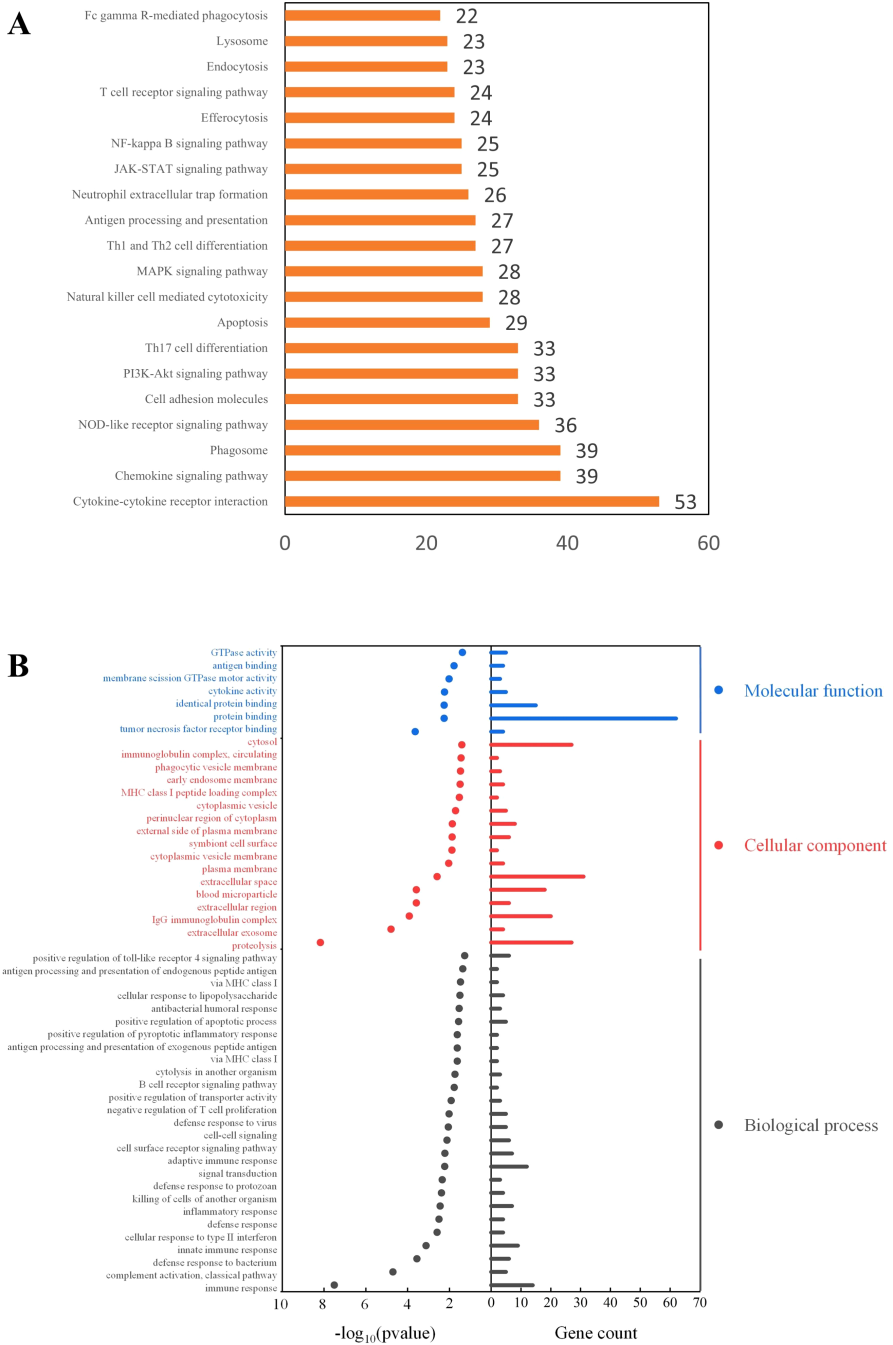
Functional enrichment analysis of M1 macrophage-associated genes. **(A)** KEGG Pathway Enrichment Analysis; **(B)** GO Enrichment Analysis.

The KEGG pathway enrichment analysis (**Figure 6A**) revealed that M1 macrophages were predominantly enriched in pathways related to cytokine signaling, chemokine signaling, and phagocytosis. The enrichment in cytokine-cytokine receptor interaction and chemokine signaling pathways suggests that M1 macrophages may recruit other immune cells by secreting cytokines and chemokines, thereby amplifying the inflammatory response. Additionally, the enrichment in the phagosome and NOD-like receptor signaling pathways indicates that M1 cells may have enhanced capacities for phagocytosis and danger signal recognition.

Gene Ontology (GO) enrichment analysis (**Figure 6B**) indicated that M1 macrophages in GLM tissues may mediate inflammatory responses through both innate and adaptive immune pathways, and regulate the immune microenvironment via signal transduction processes. Notably, GO enrichment also showed that M1 macrophages were significantly enriched in phagocytosis-related cellular components (*P* < 0.05), consistent with the findings from the KEGG pathway analysis.

### 3.7 Phagocytosis related gene expression

**Figure 7:**
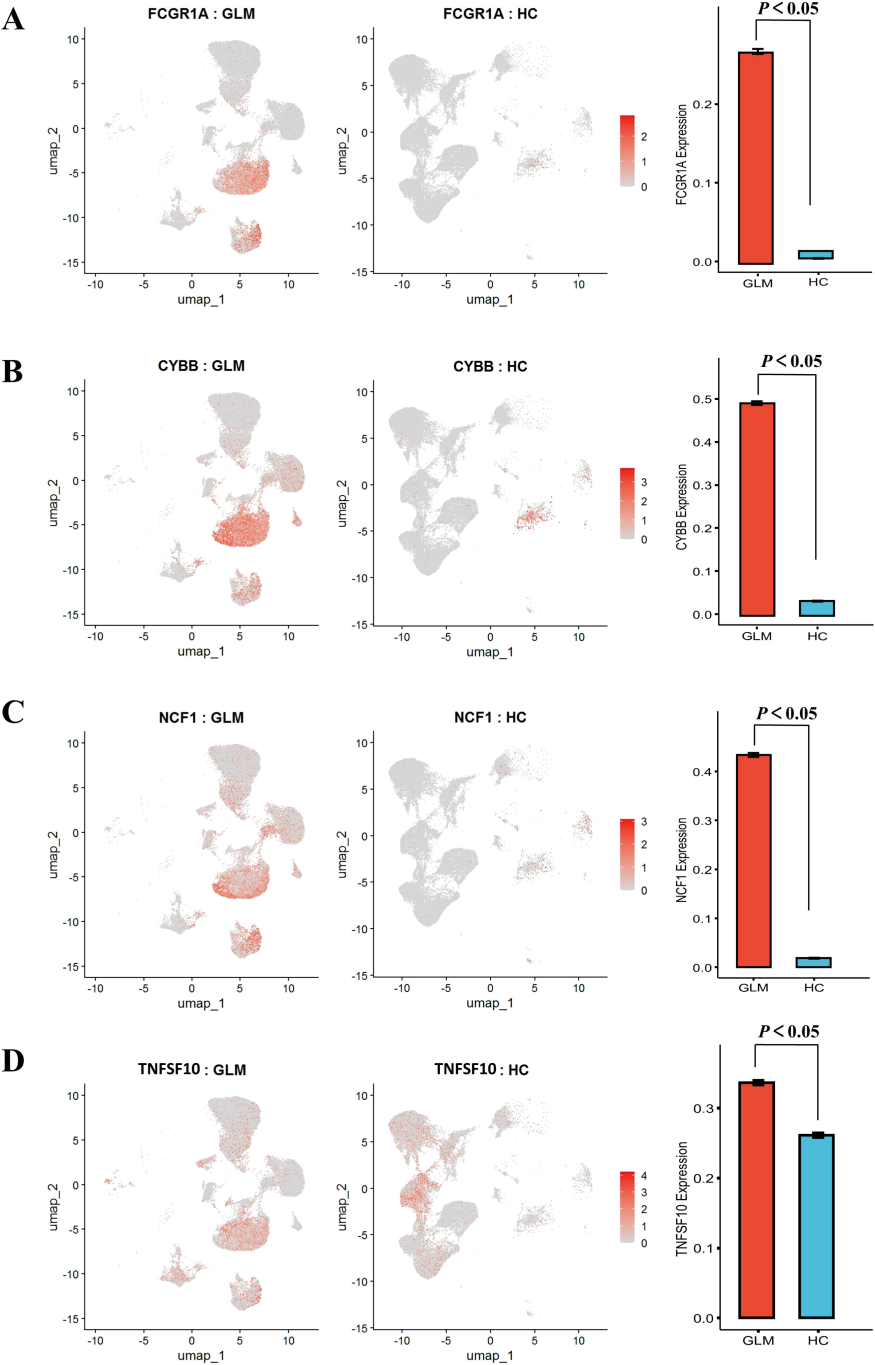
Comparative analysis of phagocytosis-related gene expression patterns with statistical validation. **(A)** Significantly Elevated Expression of FCGR1A; **(B)** Significantly Elevated Expression of CYBB; **(C)** Significantly Elevated Expression of NCF1; **(D)** Significantly Elevated Expression of TNFSF10.

To investigate phagocytic dysfunction in M1 macrophages, we focused on key components of phagosome maturation and Fcγ receptor signaling and performed t-tests. The upregulation of *FCGR1A* (CD64) (*P* < 0.05, **Figure 7A**) was accompanied by overexpression of NADPH oxidase components *CYBB* and *NCF1* (*P* < 0.05, **Figures 7B, C**), with concurrent elevation of *TNFSF10* (TRAIL) expression (*P* < 0.05, **Figure 7D**). To avoid compositional confounding from immune-cell enrichment in GLM, we compared *FCGR1A, CYBB, NCF1*, and *TNFSF10* within macrophages only. The dot plot shows higher percent expressed and higher average expression for all four genes in GLM relative to healthy controls (**Supplementary Figure S5**).

### 3.8 Intercellular communication network

By analyzing the differences in intercellular communication networks under GLM and healthy conditions, we observed two key differential features. Quantitative analysis (**Supplementary Figure S6A**) showed that the GLM group exhibited a more complex cell–cell communication network, with a significantly higher number of interactions than the healthy group (2142 vs 958). In terms of interaction strength, the GLM group displayed a much higher signaling capacity than the healthy group, with a 156% increase in average interaction strength (57.644 vs 22.505). The dual advantage in both number and strength indicates the presence of hyperactive intercellular communication in GLM lesions. The differences in the number (**Supplementary Figure S6B**) and strength (**Supplementary Figure S6C**) of interactions between different cell populations in the two groups were visualized using circle plots, in which red lines indicate enhanced signaling in the GLM group relative to the healthy control group and blue lines indicate reduced signaling, further confirming that, in the disease state of GLM, more complex communication connections are established between cells and reflecting abnormal activation of immune cells such as macrophages, T cells, and pDCs. Differential heatmap (**Supplementary Figure S6D**) analysis showed that macrophages, as a key cell type, exhibited significant enhancement in both signaling sending and receiving activities. Meanwhile, interactions between macrophages and immune cells such as T cells, pDCs, and B cells were also markedly enhanced, forming a dense immune cell communication network. Notably, stromal cells such as fibroblasts and endothelial cells also showed increased communication activity.

## 4 Discussion

Currently, the pathogenesis of GLM remains unclear. To our knowledge, this study represents the first application of single-cell RNA sequencing to investigate granulomatous mastitis at cellular resolution, providing mechanistic insights while establishing a systematic compare with healthy mammary tissue. We detected distinct immune and stromal cell populations and characterized genes involved in inflammatory responses in GLM.

The study revealed significant differences in cell composition between GLM and control groups. The GLM group was characterized by a significant enrichment of immune cells, particularly macrophages and neutrophils. Studies have shown that various stimuli, including infectious agents and foreign bodies, as well as numerous inflammatory and autoimmune diseases of unknown etiology, can promote granuloma formation (22). Some granulomas form in response to infectious agents as a mechanism to contain the infection and prevent the spread of microorganisms (23). Typically, granulomas form when macrophages cannot eliminate foreign agents, such as infectious agents or foreign bodies like self-antigens (22, 24, 25). Granuloma formation begins with the failure of phagocytosis upon the first stimulus (22). During the progression of granulomatous inflammation, macrophages transform into epithelioid and giant multinucleated cells (Langhans cells). The loss of phagocytic function leads to increased antigen expression on the surface of macrophages (22, 24). These antigens activate Th-lymphocytes, and helper T cells (Th1) release chemokines and cytokines to recruit more macrophages to the site of inflammation (26). At sites of active granuloma formation, elevated numbers of activated helper T cells stimulate B cells and secrete key pro-inflammatory cytokines (26). Cytokine overexpression exacerbates immune hyperactivity, perpetuating disease progression. In GLM, aberrant immune responses involving macrophages, T cells, B cells, and natural killer (NK) cells, together with increased secretion of pro-inflammatory cytokines, play a pivotal role in disease pathogenesis (27). While the immune mechanisms of infectious granulomas are well characterized, the factors contributing to noninfectious granulomatous inflammation remain poorly defined (28, 29).

The cellular and molecular mechanisms underlying GLM have garnered substantial attention, with immune dysregulation emerging as a key contributing factor. Zhang et al (30). reported that membrane attack complex (MAC) composed of complement localized to the epithelial membrane cells through immunoelectron microscopy, finding a significant increase in MAC expression in GLM. This increase could be an essential reason for the injury to mammary duct epithelial cells. Ucaryilmaz (31) et al. reported immune cell imbalances in GLM, including a reduced T helper lymphocyte ratio, an elevated effector T cell (Teff) ratio, and an increased natural killer (NK) cell ratio. Furthermore, regulatory B cells (Bregs) were lower in GLM patients with active disease compared to those in remission, although no significant differences were observed in Bregs and B cell subsets. Pro-­inflammatory cytokines such as IL- 6, IL- 8 and IL- 17 were upregulated in GLM patients, alongside increased serum concentrations of TNF- α, IL- 1β, IL- 2, IL- 12p70, and IL- 16 (32–34). Notably, the upregulation of IL- 1α and IL- 1β may have induced the significant increase in production of MIP- 1, including MIP- 1α (CCL3), MIP- 1β (CCL4), and MIP- 1δ (CCL15) (33, 35).

Neutrophils and macrophages exhibited the most pronounced differences between the GLM and healthy control groups. Neutrophils are inflammatory cells that infiltrate the initiation sites of inflammation and play a crucial role in innate immune responses (36). Beyond their canonical functions in innate immunity, neutrophils and macrophages play key roles in the activation and regulation of adaptive immune responses, while also bridging crosstalk between innate and adaptive immunity (37, 38).

Inflammatory stimuli trigger neutrophil activation and migration, enabling them to exert immune defense functions at lesion sites through phagocytosis, degranulation, and the formation of neutrophil extracellular traps (NETs) (39). Activated neutrophils adhere to the vascular endothelium and mediate local vascular damage and histopathological alterations via the excessive release of reactive oxygen species (ROS) and proteases (40). NETs are DNA-protein complexes released by activated neutrophils during early inflammation and are characterized by decondensed chromatin DNA backbone, citrullinated histone H3 (CitH3), and myeloperoxidase (MPO), which serve as a critical interface between innate and adaptive immunity (39).

Mechanistically, NETs induced by autoimmune stimuli activate pDCs via TLR9 and TLR7 signaling, which promotes type I IFN expression and drives autoimmune pathology (39). This antigen-presenting capacity positions NETs as amplifiers of adaptive immune responses during chronic inflammation. Excessive NETs accumulation and associated extracellular histoproteins can lead to endothelial cell damage, extracellular matrix degradation, pro-fibrotic factor release, and sustained macrophage activation, and ultimately establishing a vicious cycle (41).

Interestingly, our transcriptional data do not support a dominant involvement of NETs in GLM pathogenesis, NET-associated genes, including *MPO*, *ELANE* and *PADI4, H3C1*, were not significantly different in GLM neutrophils compared to controls. This finding suggests that NETs formation associated with neutrophil-driven inflammation is not a major feature of GLM, challenging previous studies that have linked NETs to GLM.

In addition, although both neutrophils and macrophages showed infiltration, the magnitude of macrophage expansion in GLM lesions was significantly greater than that of neutrophils compared to healthy controls, highlighting macrophages as the more prominently altered cellular population. Moreover, our comprehensive analysis of the intercellular communication network (**Supplementary Figure S6**) revealed that macrophages act as central signaling hubs. In contrast, neutrophils demonstrated limited and weak outgoing and incoming signaling interactions. In terms of the number and strength of interactions, intercellular communication by neutrophils is not extensive. Therefore, macrophages may represent a more important and informative cell type that deserves further investigation.

The high abundance and significant fold change of macrophages in the GLM group support the hypothesis that macrophages play a pivotal role in the pathogenesis of GLM (42). Studies have shown that the infiltration and proliferation of macrophages are directly responsible for inducing granuloma development and play a crucial role in GLM by releasing inflammatory factors and modulating the M1/M2 phenotype (29, 43). Macrophages are key components of the innate immune system, responsible for eliminating foreign substances through phagocytosis (24, 44). Defective phagocytic activity of macrophages initiates granulomatous inflammation (28, 29, 44). Whole-exome sequencing of GLM revealed that most identified variants are enriched in genes involved in innate immunity, particularly those regulating macrophage activity and phagocytic processes (45). However, our study revealed that macrophages not only contribute to innate immunity but also play a critical role in orchestrating adaptive immune responses within the context of GLM.

Macrophage activation states are classically categorized into pro-inflammatory M1 and anti-inflammatory M2 phenotypes based on their interaction with helper T (Th) cells (46), a process termed macrophage polarization. M1 macrophages, activated by Th1 cells, mediate Th1-type immune responses by releasing pro-inflammatory cytokines that drive early tissue injury and inflammation (47). In contrast, M2 macrophages, induced by Th2-derived cytokines, exert anti-inflammatory functions by secreting regulatory cytokines, growth, and repair factors, thereby facilitating immune tolerance, tissue regeneration, and, in some contexts, tumor progression (48). M2a macrophages assist in tissue remodeling, and immune regulation. M2b macrophages, which are activated by immune complexes and TLR signaling, as well as IL1R activation, release factors that exert an inhibitory effect on the immune response. M2c macrophages are involved in phagocytosis and angiogenesis through the scavenging of pro-inflammatory factors. M2d macrophages, derived from polarized M1 macrophages, are anti-inflammatory and angiogenic, with increasing levels of extracellular adenosine reported to be a key factor in M2d polarization (49).

As shown in **Supplementary Figure S4**, M1 macrophages are the predominant subtype in both groups—accounting for 64.5% in GLM and 73.1% in healthy breast tissue. M2 subtypes are present in both groups but at lower proportions (GLM: M2a 5.1%, M2b 10.1%, M2c 20.3%; Healthy: M2a 9.6%, M2b 3.8%, M2c 13.5%). While M1 macrophages remain the numerical majority in both groups, our data also indicate meaningful shifts within M2 macrophages. M2 macrophages, induced by Th2-derived cytokines, exert anti-inflammatory functions by secreting regulatory cytokines, growth, and repair factors, thereby facilitating immune tolerance, tissue regeneration. In GLM, M2b and M2c are selectively expanded, suggesting active immunologic reprogramming of the tissue microenvironment. Transcriptional features are consistent with this interpretation: M2b macrophages exhibited high expression of *MERTK* and *IGF1*, suggesting potential involvement in anti-inflammatory signaling regulation and cell survival. In contrast, M2c macrophages showed elevated expression of *COL1A1*, indicating a possible role in collagen synthesis and tissue repair. Together, these changes are compatible with the transition from acute to chronic granulomatous inflammation, in which persistent stimuli drive parallel pro-inflammatory (M1) and resolving (M2) programs within the macrophage compartment.

During postnatal mammary development, macrophages are recruited to the terminal end buds, where they play key roles in sensing tissue damage and preserving homeostasis (42, 48, 50). Under physiological conditions, resident macrophages maintain a dynamic balance; however, pathological stimuli may disrupt this equilibrium, leading to the dominance of a specific subtype—a shift known as M1/M2 drift—which may contribute to the initiation and progression of GLM (42). KEGG enrichment analysis showed that M1 macrophages were primarily enriched in pathways related to cytokine signaling, chemokine signaling, and phagocytosis. Similarly, GO enrichment analysis revealed significant enrichment of M1 macrophages in phagocytosis-related cellular components (*P* < 0.05), aligning with the KEGG results. Previous studies have reported pathogenic variants in genes associated with macrophage function and phagocytosis—such as *FCGR1A*, *MPO*, *FV*, *PROC*, and *IFI30*—in GLM patients (45), further supporting the hypothesis that the phagosome pathway may play a crucial role in the pathogenesis of GLM.

Notably, our single-cell RNA sequencing data revealed that macrophages in GLM patients exhibited elevated expression of *FCGR1A* (CD64), the high-affinity IgG receptor, thereby enhancing phagocytic activity and activating the NADPH oxidase pathway. Expression levels of NADPH oxidase complex genes *CYBB* and *NCF1* were significantly upregulated. Upon antigen uptake, downstream signaling cascades lead to the phosphorylation of cytosolic components—particularly *NCF1 (* 51) —which cooperatively activate *NOX2* and trigger a robust inflammatory immune response (52). NADPH oxidase-derived reactive oxygen species (ROS) have been demonstrated to upregulate *TNFSF10* (TRAIL) expression (53), a finding that aligns with the observed co-upregulation of NADPH oxidase core components (*CYBB*/*NCF1*) and *TNFSF10* (TRAIL) in our study. TRAIL could affect the activity of NF-κB and the expression of its downstream proinflammatory cytokines IL-1β, IL-6, and TNF-α in macrophages (21), thereby exacerbating the inflammatory response (54–56). The association between IL-6, TNF-α, and GLM has been confirmed by multiple studies. Serum levels of pro-inflammatory cytokines such as IL-6 and TNF-α are significantly elevated in GLM patients (55, 57).

M1 macrophages can activate multiple inflammatory signaling pathways, including the NADPH pathway, to generate nitric oxide (NO) and ROS. These molecules, in turn, activate downstream TRAIL, stimulating the secretion of pro-inflammatory cytokines such as IL-6 and TNF-α, thereby exacerbating the inflammatory response. Consequently, regulating the CD64 - NADPH - TRAIL signaling pathway in macrophages may be a key strategy to mitigate the inflammatory pathogenesis of GLM. A recent study by Min et al. demonstrated that the use of anti-CD64 antibodies inhibited anti-citrullinated protein antibody induced osteoclastogenesis in rheumatoid arthritis (RA) patients (58). Similarly, Liu et al. showed that Sinomenine (SIN), an anti-inflammatory drug used in RA treatment, attenuated CD64+ macrophages in synovial tissue and CD11b+F4/80+CD64+ resident macrophages in RA tissues and lymphoid organs, further supporting CD64 as a therapeutic target in RA (59). These findings further bolster our hypothesis that the CD64-NADPH-TRAIL axis could be a promising therapeutic target for GLM, particularly in the context of macrophage-driven inflammation.

Similarly, the significant upregulation of MHC II in GLM macrophages suggests an enhanced antigen presentation function. In adaptive immune responses, major histocompatibility complex class II (MHC II) molecules present antigens to CD4^+^ T cells. Under the regulation of co-stimulatory signals and cytokines, this process induces their differentiation into various Th subsets. M1 macrophages are activated by Th1 cells, contributing to the Th1 immune response and the release of pro-inflammatory cytokines. Antigen-presenting cells (APCs), including dendritic cells, macrophages, and B cells, uptake and process antigens, loading their peptide fragments onto MHC II molecules for presentation on the cell surface (60). During phagosome maturation, macrophages process engulfed antigens into peptide fragments, which are then presented to CD4^+^ T cells via MHC II molecules. CD4^+^ T cells recognize MHC II-antigen complexes through their T cell receptor (TCR), with CD4 molecules enhancing signal transduction to promote T cell activation. During this process, co-stimulatory signals provided by APCs and cytokines from the local microenvironment determine the differentiation direction of CD4^+^ T cells (61). Th cells, particularly follicular helper T (Tfh) cells, play a crucial role in the differentiation of B cells into plasma cells (62). Within the germinal center, Tfh cells guide B cells through CD40-CD40L interactions and IL-21 signaling, driving affinity maturation and class switching. This process ultimately results in the differentiation of B cells into plasma cells responsible for secreting high-affinity antibodies (63). These IgG antibodies then enhance phagocytosis via Fcγ receptors (FcγR).

This study has certain limitations. The sample size of this study is relatively small, based only on 3 GLM samples and 3 healthy control samples, which may limit its universality. Despite this, the high consistency in cell composition and key pathogenic pathways among the three GLM patients in this study strongly supports the reliability of our core findings. Future research needs to verify the universality and heterogeneity of these cell subpopulations in larger cohorts. Moreover, the findings presented here are predominantly derived from transcriptional data. In the future, we need to analyze larger samples of GLM and healthy tissues at the protein level.

## 5 Conclusion

To our knowledge, this is the first scRNA-seq study of GLM, identifying 11 major cellular populations and implicating macrophages—especially M1 subtype—as central to disease immunopathology. We report dysregulated expression of CD64, NADPH oxidase components, and TRAIL, prompting the hypothesis that phagocytic function may be impaired and nominating this axis as a potential therapeutic target.

## Data Availability

The raw data supporting this study have been deposited in the Gene Expression Omnibus (GEO) Database under the permanent accession number: GSE302333.

## Supplementary material

Supplementary Figure 1 Gene expression and clustering of macrophage clusters. **(A)** Dot Plot of Gene Expression Across Macrophage Clusters; **(B)** UMAP Plot of Macrophage Clusters.

Supplementary Figure 2 Expression of myoepithelial markers.

Supplementary Figure 3 T Cell Subtype Annotation.

Supplementary Figure 4 Comparison of macrophage subtypes between GLM and HC groups. **(A)** Bar Plot of Macrophage Subtypes; **(B)** Stacked Bar Plot of Macrophage Subtypes.

Supplementary Figure 5 Group-wise expression of FCGR1A, CYBB, NCF1, and TNFSF10 in macrophages.

Supplementary Figure 6 Intercellular communication network between GLM and healthy tissues. **(A)** Quantification of the Number of Inferred Interactions and Interaction Strength; **(B)** Number of Interactions Between Cell Populations; **(C)** Interaction Strength Between Cell Populations; **(D)** Differential Heatmap of Outgoing and Incoming Signaling Across Cell Populations.

Supplementary Table 1 T cell subtype marker genes.

## References

[1] Liu R Luo Z Dai C Wei Y Yan S Kuang X. Corynebacterium parakroppenstedtii secretes a novel glycolipid to promote the development of granulomatous lobular mastitis. Signal Transduct Target Ther. (2024) 9:292. doi: 10.1038/s41392-024-01984-0, PMID: 39428541 PMC11491465

[2] Chinese Society of Pathology Breast Pathology Group Tumor Pathology Committee of China Anti-Cancer Association. Chinese expert consensus on the pathological diagnosis of granulomatous lobular mastitis (2024 version). Zhonghua Bing Li Xue Za Zhi. (2024) 53:996–1004. doi: 10.3760/cma.j.cn112151-20240612-00382, PMID: 39375079

[3] Zeng Y Zhang D Zhao W Fu N Huang Q Li S. Predisposing factors for granulomatous lobular mastitis: A case-control study. Int J Womens Health. (2023) 15:1063–75. doi: 10.2147/IJWH.S414054, PMID: 37795195 PMC10547110

[4] Zeng Y Zhang D Fu N Zhao W Huang Q Cui J. Risk factors for granulomatous mastitis and establishment and validation of a clinical prediction model (Nomogram). Risk Manag Healthc Policy. (2023) 16:2209–22. doi: 10.2147/RMHP.S431228, PMID: 37881167 PMC10596285

[5] Zeng Y Wang M Gao X Zhang D Fu N Zhao W. Clinical characteristics of patients with granulomatous lobular mastitis associated with Corynebacterium parakroppenstedtii infection and drug sensitivity analysis of the isolated strains. Ann Clin Microbiol Antimicrob. (2024) 23:95. doi: 10.1186/s12941-024-00755-7, PMID: 39472981 PMC11520474

[6] Wang M Zhang D Fu N Liu M Zhang H Feng S. Clinical features of cystic neutrophil granulomatous mastitis in 62 cases. Heliyon. (2025) 11:e42415. doi: 10.1016/j.heliyon.2025.e42415, PMID: 39991218 PMC11847053

[7] Wang M Liu Y Zhang D Fu N Wang Y Liu M. Analysis of the clinical characteristics and ultrasonographic features in 141 cases of cystic neutrophilic granulomatous mastitis. Acad Radiol. (2025) 32:2489–96. doi: 10.1016/j.acra.2024.12.034, PMID: 39753477

[8] Wang M Zeng Y Liu M Zhang D Zhao D Wang J. Rat model of cystic neutrophilic granulomatous mastitis by corynebacterium kroppenstedtii. J Inflammation Res. (2025) 18:1887–98. doi: 10.2147/JIR.S500310, PMID: 39931172 PMC11809361

[9] Yuan Q-Q Xiao S-Y Farouk O Du Y-T Sheybani F Tan QT. Management of granulomatous lobular mastitis: an international multidisciplinary consensus (2021 edition). Mil Med Res. (2022) 9:20. doi: 10.1186/s40779-022-00380-5, PMID: 35473758 PMC9040252

[10] Alkon N Bauer WM Krausgruber T Goh I Griss J Nguyen V. Single-cell analysis reveals innate lymphoid cell lineage infidelity in atopic dermatitis. J Allergy Clin Immunol. (2022) 149:624–39. doi: 10.1016/j.jaci.2021.07.025, PMID: 34363841 PMC9130781

[11] Binvignat M Miao BY Wibrand C Yang MM Rychkov D Flynn E. Single-cell RNA-Seq analysis reveals cell subsets and gene signatures associated with rheumatoid arthritis disease activity. JCI Insight. (2024) 9:e178499. doi: 10.1172/jci.insight.178499, PMID: 38954480 PMC11343607

[12] Qi J Sun H Zhang Y Wang Z Xun Z Li Z. Single-cell and spatial analysis reveal interaction of FAP+ fibroblasts and SPP1+ macrophages in colorectal cancer. Nat Commun. (2022) 13:1742. doi: 10.1038/s41467-022-29366-6, PMID: 35365629 PMC8976074

[13] Zhang L Li Z Skrzypczynska KM Fang Q Zhang W O’Brien SA. Single-cell analyses inform mechanisms of myeloid-targeted therapies in colon cancer. Cell. (2020) 181:442–459.e29. doi: 10.1016/j.cell.2020.03.048, PMID: 32302573

[14] Liu W-B Huang G-R Liu B-L Hu H-K Geng J Rui H-L. Single cell landscape of parietal epithelial cells in healthy and diseased states. Kidney Int. (2023) 104:108–23. doi: 10.1016/j.kint.2023.03.036, PMID: 37100348

[15] Zheng GXY Terry JM Belgrader P Ryvkin P Bent ZW Wilson R. Massively parallel digital transcriptional profiling of single cells. Nat Commun. (2017) 8:14049. doi: 10.1038/ncomms14049, PMID: 28091601 PMC5241818

[16] Hao Y Hao S Andersen-Nissen E Mauck WM 3rd Zheng S Butler A. Integrated analysis of multimodal single-cell data. Cell. (2021) 184:3573–3587.e29. doi: 10.1016/j.cell.2021.04.048, PMID: 34062119 PMC8238499

[17] Germain P-L Sonrel A Robinson MD. PipeComp, a general framework for the evaluation of computational pipelines, reveals performant single cell RNA-seq preprocessing tools. Genome Biol. (2020) 21:227. doi: 10.1186/s13059-020-02136-7, PMID: 32873325 PMC7465801

[18] Ma X Yang A Fan X Liu H Gu Y Wang Z. Resistin alleviates lipopolysaccharide-induced inflammation in bovine alveolar macrophages by activating the AMPK/mTOR signaling pathway and autophagy. Heliyon. (2024) 10:e38026. doi: 10.1016/j.heliyon.2024.e38026, PMID: 39386884 PMC11462211

[19] Zhan W Luo W Zhang Y Xiang K Chen X Shen S. Sputum transcriptomics reveals FCN1+ Macrophage activation in mild eosinophilic asthma compared to non-asthmatic eosinophilic bronchitis. Allergy Asthma Immunol Res. (2024) 16:55–70. doi: 10.4168/aair.2024.16.1.55, PMID: 38262391 PMC10823142

[20] Zhan C Zhou Z Huang Y Huang S Lin Z He F. Exploration of the shared gene signatures and molecular mechanisms between periodontitis and inflammatory bowel disease: evidence from transcriptome data. Gastroenterol Rep (Oxf). (2023) 11:goad041. doi: 10.1093/gastro/goad041, PMID: 37456714 PMC10348870

[21] Gao J Wang D Liu D Liu M Ge Y Jiang M. Tumor necrosis factor-related apoptosis-inducing ligand induces the expression of proinflammatory cytokines in macrophages and re-educates tumor-associated macrophages to an antitumor phenotype. Mol Biol Cell. (2015) 26:3178–89. doi: 10.1091/mbc.E15-04-0209, PMID: 26224317 PMC4569310

[22] Pagán AJ Ramakrishnan L. The formation and function of granulomas. Annu Rev Immunol. (2018) 36:639–65. doi: 10.1146/annurev-immunol-032712-100022, PMID: 29400999

[23] Shah KK Pritt BS Alexander MP. Histopathologic review of granulomatous inflammation. J Clin Tuberc Other Mycobact Dis. (2017) 7:1–12. doi: 10.1016/j.jctube.2017.02.001, PMID: 31723695 PMC6850266

[24] Yang S Zhao M Jia S. Macrophage: Key player in the pathogenesis of autoimmune diseases. Front Immunol. (2023) 14:1080310. doi: 10.3389/fimmu.2023.1080310, PMID: 36865559 PMC9974150

[25] Petersen HJ Smith AM. The role of the innate immune system in granulomatous disorders. Front Immunol. (2013) 4:120. doi: 10.3389/fimmu.2013.00120, PMID: 23745122 PMC3662972

[26] Yang H Liu H Zheng Y Li B Wang S Zhang J. Cornus officinalis total glycosides alleviate granulomatous lobular mastitis via the B7-CD28/CTLA-4 costimulatory pathway. Chem Biodivers. (2025) 22:e202401539. doi: 10.1002/cbdv.202401539, PMID: 39344790

[27] Lou Y Xu H Lu Z Wang B Liu X. Immune regulation: a new strategy for traditional Chinese medicine-based treatment of granulomatous lobular mastitis. Front Immunol. (2024) 15:1494155. doi: 10.3389/fimmu.2024.1494155, PMID: 39544943 PMC11560460

[28] Lee H-J Woo Y Hahn T-W Jung YM Jung Y-J. Formation and maturation of the phagosome: A key mechanism in innate immunity against intracellular bacterial infection. Microorganisms. (2020) 8:1298. doi: 10.3390/microorganisms8091298, PMID: 32854338 PMC7564318

[29] Wilson JL Mayr HK Weichhart T. Metabolic programming of macrophages: implications in the pathogenesis of granulomatous disease. Front Immunol. (2019) 10:2265. doi: 10.3389/fimmu.2019.02265, PMID: 31681260 PMC6797840

[30] Zhang H-J Ding P-P Zhang X-S Wang X-C Sun D-W Bu Q-A. MAC mediates mammary duct epithelial cell injury in plasma cell mastitis and granulomatous mastitis. Int Immunopharmacol. (2022) 113:109303. doi: 10.1016/j.intimp.2022.109303, PMID: 36252469

[31] Ucaryilmaz H Koksal H Emsen A Kadoglou N Dixon JM Artac H. The role of regulatory T and B cells in the etiopathogenesis of idiopathic granulomatous mastitis. Immunol Invest. (2022) 51:357–67. doi: 10.1080/08820139.2020.1832114, PMID: 33034215

[32] Koksal H Vatansev H Artac H Kadoglou N. The clinical value of interleukins-8, -10, and -17 in idiopathic granulomatous mastitis. Clin Rheumatol. (2020) 39:1671–7. doi: 10.1007/s10067-020-04925-8, PMID: 31916110

[33] Li F Nie L Huang J Sin T-H Wang X Zhang F. Evaluation of significantly changed chemokine factors of idiopathic granulomatous mastitis in non-puerperal patients. FASEB J. (2024) 38:e23745. doi: 10.1096/fj.202400114RRR, PMID: 38923065

[34] Esmaeil NK Salih AM Hammood ZD Pshtiwan LRA Abdullah AM Kakamad FH. Clinical, microbiological, immunological and hormonal profiles of patients with granulomatous mastitis. BioMed Rep. (2023) 18:41. doi: 10.3892/br.2023.1624, PMID: 37325183 PMC10265128

[35] Korbecki J Kojder K Simińska D Bohatyrewicz R Gutowska I Chlubek D. CC chemokines in a tumor: A review of pro-cancer and anti-cancer properties of the ligands of receptors CCR1, CCR2, CCR3, and CCR4. Int J Mol Sci. (2020) 21:8412. doi: 10.3390/ijms21218412, PMID: 33182504 PMC7665155

[36] Jung YJ Lee AS Nguyen-Thanh T Kim D Kang KP Lee S. SIRT2 regulates LPS-induced renal tubular CXCL2 and CCL2 expression. J Am Soc Nephrol. (2015) 26:1549–60. doi: 10.1681/ASN.2014030226, PMID: 25349202 PMC4483578

[37] Jaillon S Ponzetta A Mitri DD Santoni A Bonecchi R Mantovani A. Neutrophil diversity and plasticity in tumour progression and therapy. Nat Rev Cancer. 20:485–503. doi: 10.1038/s41568-020-0281-y, PMID: 32694624

[38] Gieseck RL 3rd Wilson MS Wynn TA. Type 2 immunity in tissue repair and fibrosis. Nat Rev Immunol. (2018) 18:62–76. doi: 10.1038/nri.2017.90, PMID: 28853443

[39] Papayannopoulos V. Neutrophil extracellular traps in immunity and disease. Nat Rev Immunol. (2018) 18:134–47. doi: 10.1038/nri.2017.105, PMID: 28990587

[40] Segel GB Halterman MW Lichtman MA. The paradox of the neutrophil’s role in tissue injury. J Leukoc Biol. (2011) 89:359–72. doi: 10.1189/jlb.0910538, PMID: 21097697 PMC6608002

[41] Krishnamoorthy N Douda DN Brüggemann TR Ricklefs I Duvall MG Abdulnour R-EE. Neutrophil cytoplasts induce T(H)17 differentiation and skew inflammation toward neutrophilia in severe asthma. Sci Immunol. (2018) 3:eaao4747. doi: 10.1126/sciimmunol.aao4747, PMID: 30076281 PMC6320225

[42] Xie L Feng J Gao Q Qu W Shao S Sun J. The autoimmune profiles in the etiopathogenesis of granulomatous lobular mastitis. Immunobiology. (2025) 230:152878. doi: 10.1016/j.imbio.2025.152878, PMID: 39922144

[43] Xing Z Afkhami S Bavananthasivam J Fritz DK D’Agostino MR Vaseghi-Shanjani M. Innate immune memory of tissue-resident macrophages and trained innate immunity: Re-vamping vaccine concept and strategies. J Leukoc Biol. (2020) 108:825–34. doi: 10.1002/JLB.4MR0220-446R, PMID: 32125045

[44] Saferding V Blüml S. Innate immunity as the trigger of systemic autoimmune diseases. J Autoimmun. (2020) 110:102382. doi: 10.1016/j.jaut.2019.102382, PMID: 31883831

[45] Ozer L Koksal H. Whole exome sequencing for identifying rare genetic variants related to idiopathic granulomatous mastitis. Clin Rheumatol. (2025) 44:143–1850. doi: 10.1007/s10067-025-07343-w, PMID: 39992598 PMC11993501

[46] Mosser DM Hamidzadeh K Goncalves R. Macrophages and the maintenance of homeostasis. Cell Mol Immunol. (2021) 18:579–87. doi: 10.1038/s41423-020-00541-3, PMID: 32934339 PMC7491045

[47] Hu Q Lyon CJ Fletcher JK Tang W Wan M Hu TY. Extracellular vesicle activities regulating macrophage- and tissue-mediated injury and repair responses. Acta Pharm Sin B. (2021) 11:1493–512. doi: 10.1016/j.apsb.2020.12.014, PMID: 34221864 PMC8245807

[48] Kong C Zhang C Wu Y Zeng Z Yu H Zeng J. The expression and meaning of CD68, CD163, CD57, and IgG4 in granulomatous lobular mastitis. Gland Surg. (2020) 9:936–49. doi: 10.21037/gs-20-419, PMID: 32953603 PMC7475371

[49] Sezginer O Unver N. Dissection of pro-tumoral macrophage subtypes and immunosuppressive cells participating in M2 polarization. Inflammation Res. (2024) 73:1411–23. doi: 10.1007/s00011-024-01907-3, PMID: 38935134 PMC11349836

[50] Yunna C Mengru H Lei W Weidong C. Macrophage M1/M2 polarization. Eur J Pharmacol. (2020) 877:173090. doi: 10.1016/j.ejphar.2020.173090, PMID: 32234529

[51] Kuihon SVNP Sevart BJ Abbey CA Bayless KJ Chen B. The NADPH oxidase 2 subunit p47(phox) binds to the WAVE regulatory complex and p22(phox) in a mutually exclusive manner. J Biol Chem. (2024) 300:107130. doi: 10.1016/j.jbc.2024.107130, PMID: 38432630 PMC10979099

[52] Aimeur S Fas BA Serfaty X Santuz H Sacquin-Mora S Bizouarn T. Structural profiles of the full phagocyte NADPH oxidase unveiled by combining computational biology and experimental knowledge. J Biol Chem. (2024) 300:107943. doi: 10.1016/j.jbc.2024.107943, PMID: 39481598 PMC11647612

[53] Ibraheem K Yhmed AMA Nasef MM Georgopoulos NT. TRAF3/p38-JNK signalling crosstalk with intracellular-TRAIL/caspase-10-induced apoptosis accelerates ROS-driven cancer cell-specific death by CD40. Cells. (2022) 11:3274. doi: 10.3390/cells11203274, PMID: 36291141 PMC9600997

[54] He H Jiang T Ding M Zhu Y Xu X Huang Y. Nox1/PAK1 is required for angiotensin II-induced vascular inflammation and abdominal aortic aneurysm formation. Redox Biol. (2025) 79:103477. doi: 10.1016/j.redox.2024.103477, PMID: 39721498 PMC11732235

[55] Li J Zeng Y Wang M Liu Y Guo Y Zhao W. Immune markers and inflammatory cytokines in granulomatous lobular mastitis: A case-control study. J Inflamm Res. (2024) 17:8647–8657. doi: 10.2147/JIR.S492464, PMID: 39553312 PMC11566206

[56] Barnabei L Laplantine E Mbongo W Rieux-Laucat F Weil R. NF-κB: at the borders of autoimmunity and inflammation. Front Immunol. (2021) 12:716469. doi: 10.3389/fimmu.2021.716469, PMID: 34434197 PMC8381650

[57] Zhou Y Wu J Ma L Wang B Meng T Chen H. Differences and significance of peripheral blood interleukin-6 expression between patients with granulomatous lobular mastitis and those with benign breast tumors. Front Med (Lausanne). (2023) 10:1273406. doi: 10.3389/fmed.2023.1273406, PMID: 37817809 PMC10561106

[58] Min HK Lee JY Lee SH Ju JH Kim HR. Suppressing anti-citrullinated protein antibody-induced osteoclastogenesis in rheumatoid arthritis using anti-CD64 and PAD-2 inhibitors. Clin Exp Rheumatol. (2025) 43:79–86. doi: 10.55563/clinexprheumatol/d9iizz, PMID: 39152765

[59] Liu W Zhang Y Zhu W Ma C Ruan J Long H. Sinomenine inhibits the progression of rheumatoid arthritis by regulating the secretion of inflammatory cytokines and monocyte/macrophage subsets. Front Immunol. (2018) 9:2228. doi: 10.3389/fimmu.2018.02228, PMID: 30319663 PMC6168735

[60] Neefjes J Jongsma MLM Paul P Bakke O. Towards a systems understanding of MHC class I and MHC class II antigen presentation. Nat Rev Immunol. (2011) 11:823–36. doi: 10.1038/nri3084, PMID: 22076556

[61] O’Shea JJ Paul WE. Mechanisms underlying lineage commitment and plasticity of helper CD4+ T cells. Science. (2010) 327:1098–102. doi: 10.1126/science.1178334, PMID: 20185720 PMC2997673

[62] Crotty S. Follicular helper CD4 T cells (TFH). Annu Rev Immunol. (2011) 29:621–63. doi: 10.1146/annurev-immunol-031210-101400, PMID: 21314428

[63] Victora GD Nussenzweig MC. Germinal centers. Annu Rev Immunol. (2012) 30:429–57. doi: 10.1146/annurev-immunol-020711-075032, PMID: 22224772

