# Supplementary figures and images for: Single-cell RNA sequencing analysis reveals cell landscape and gene signatures associated with granulomatous lobular mastitis
Source: Front Immunol. 2025 Oct 16;16:1624640. doi: 10.3389/fimmu.2025.1624640 (PMC12571661; doi:10.3389/fimmu.2025.1624640)

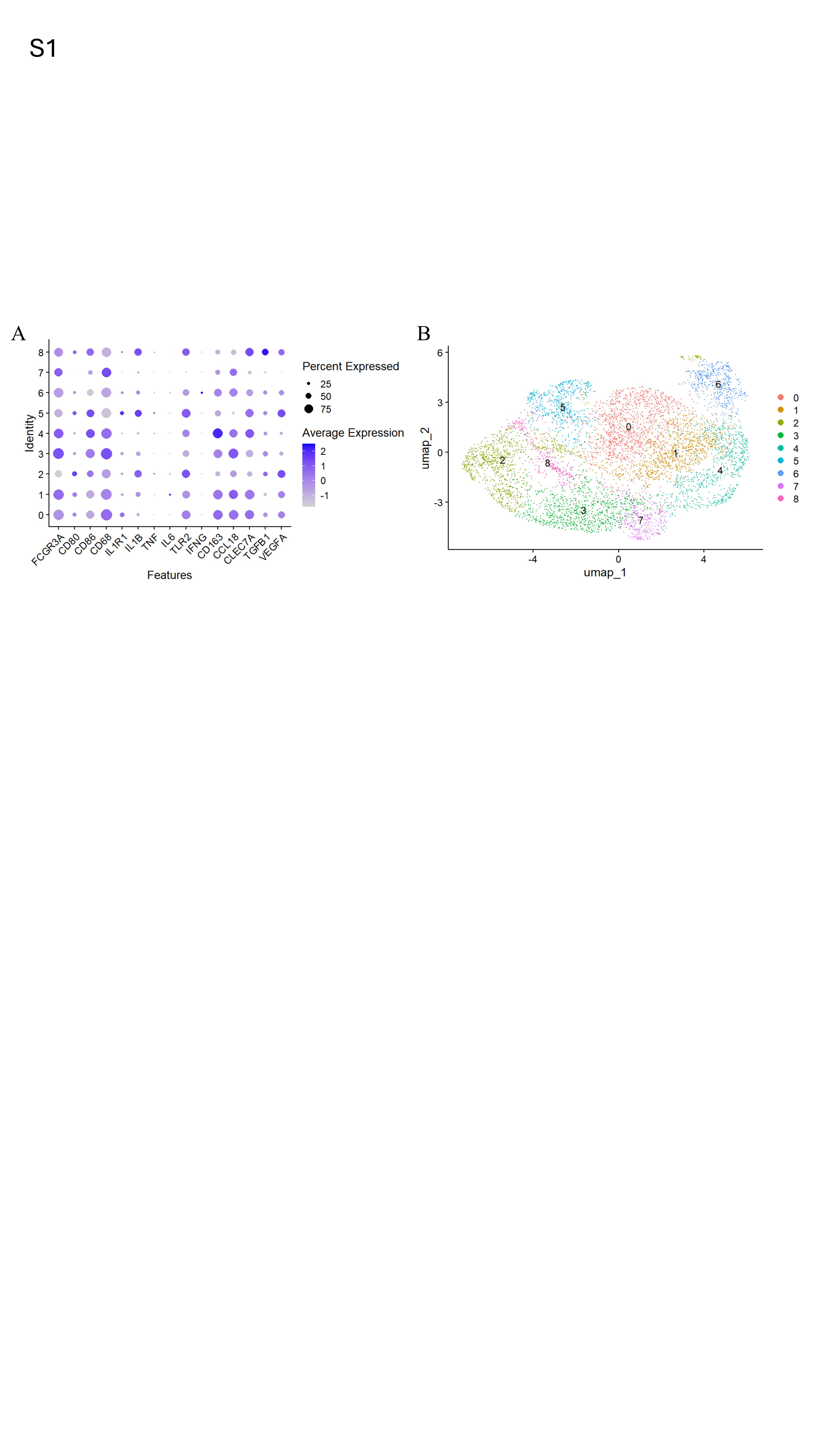

Supplement: Supplementary Figure 1 — Gene expression and clustering of macrophage clusters. (A) Dot Plot of Gene Expression Across Macrophage Clusters; (B) UMAP Plot of Macrophage Clusters. [file Image1.jpeg]

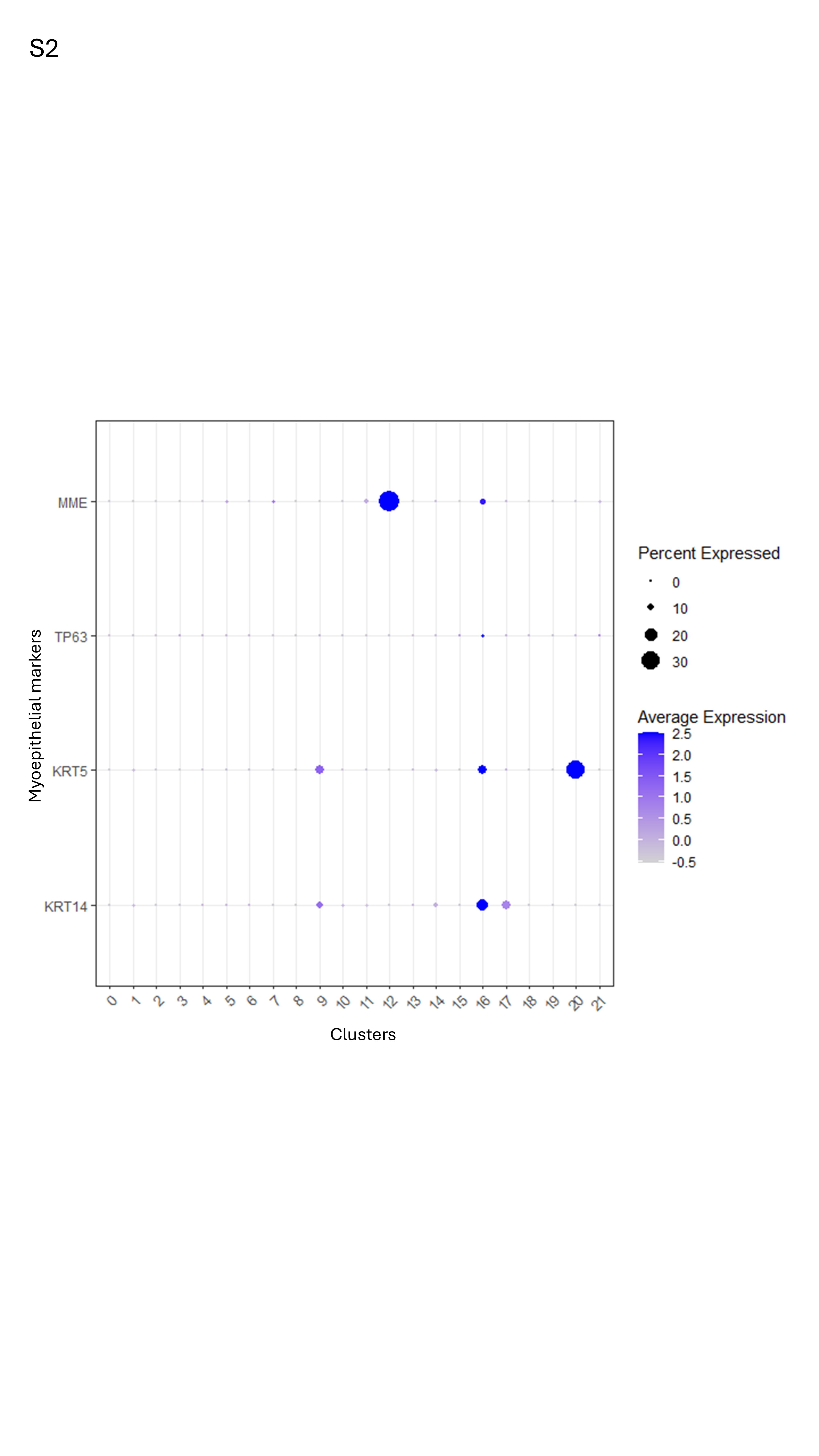

Supplement: Supplementary Figure 2 — Expression of myoepithelial markers. [file Image2.jpeg]

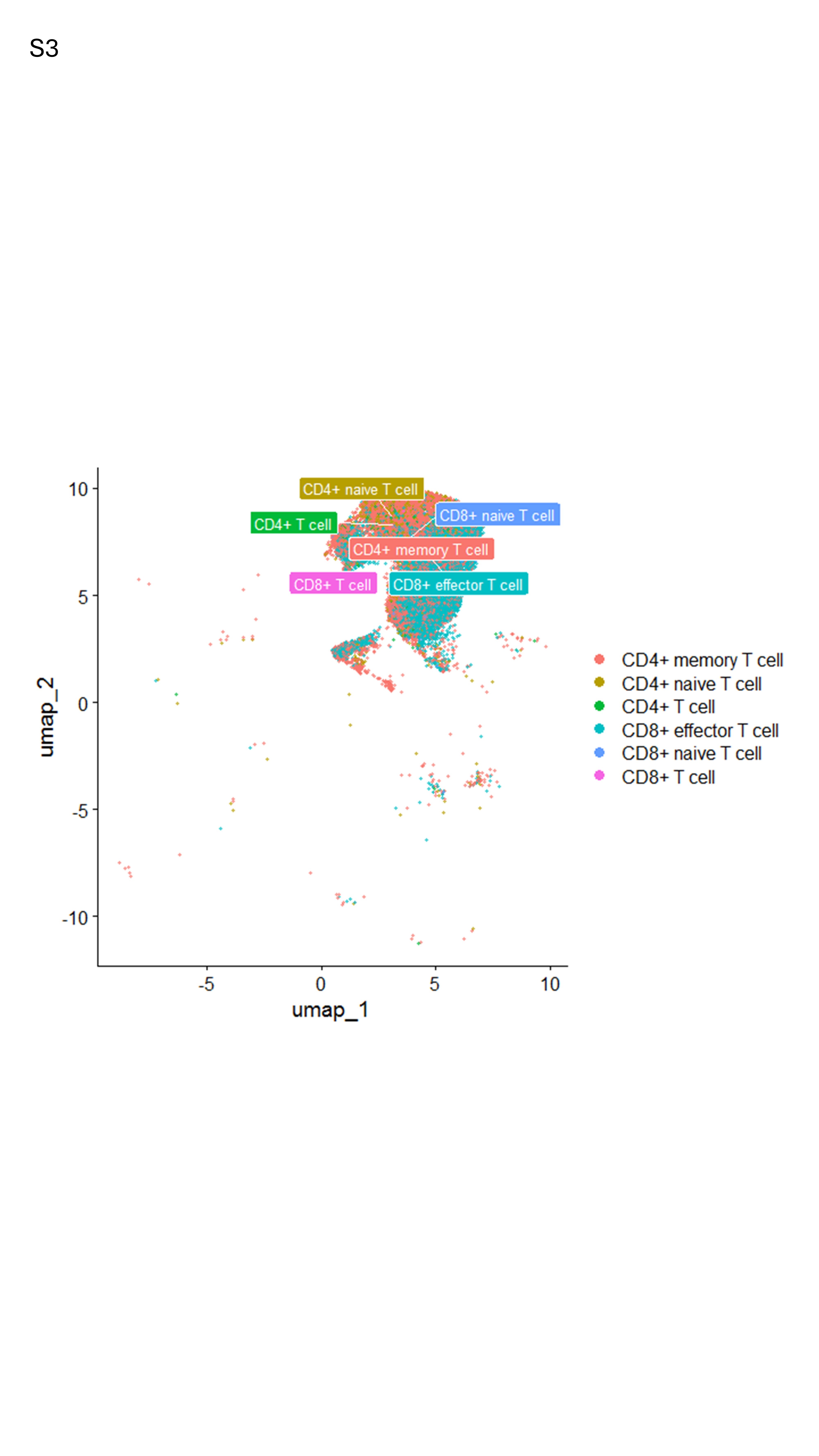

Supplement: Supplementary Figure 3 — T Cell Subtype Annotation. [file Image3.jpeg]

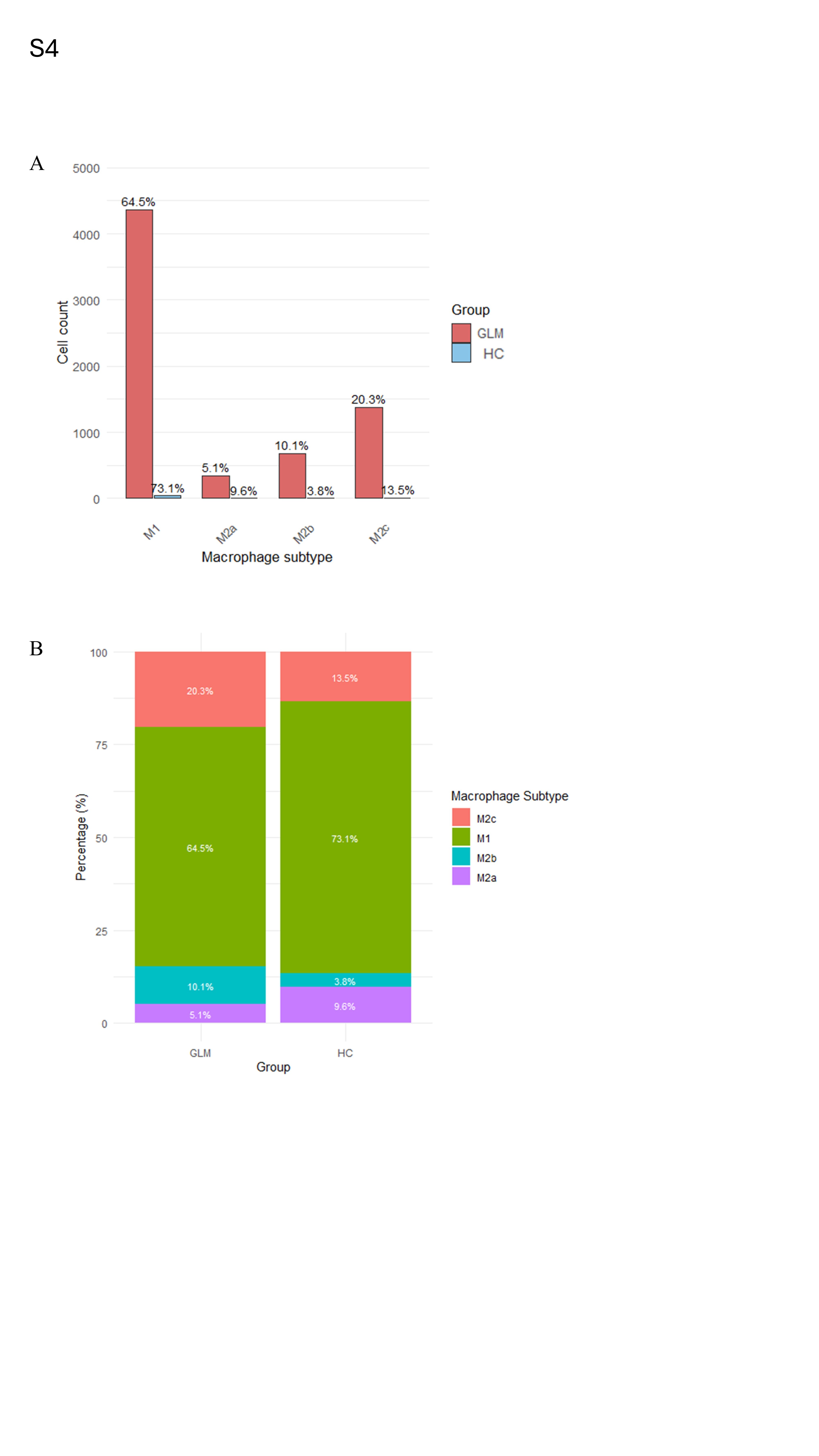

Supplement: Supplementary Figure 4 — Comparison of macrophage subtypes between GLM and HC groups. (A) Bar Plot of Macrophage Subtypes; (B) Stacked Bar Plot of Macrophage Subtypes. [file Image4.jpeg]

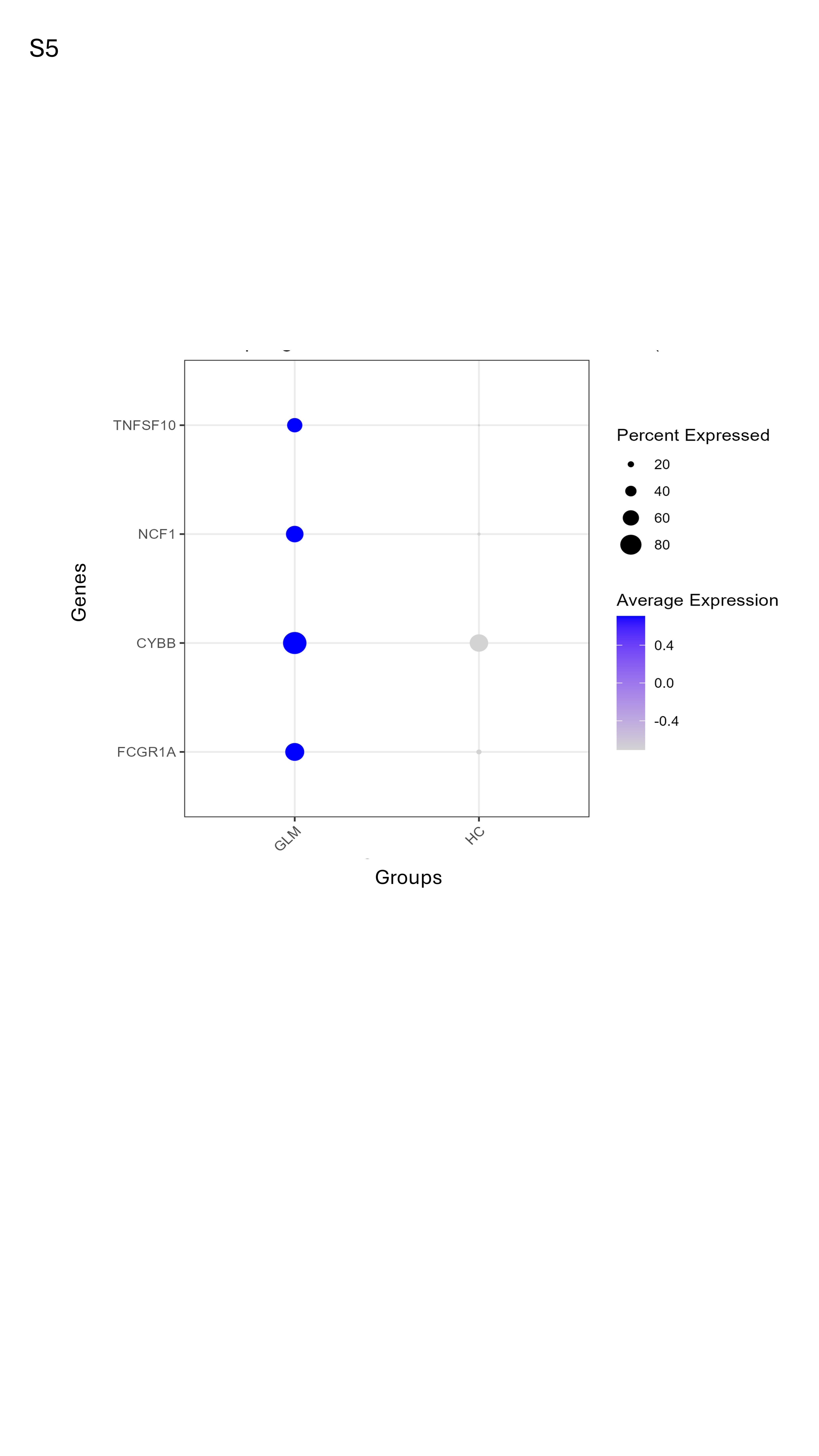

Supplement: Supplementary Figure 5 — Group-wise expression of FCGR1A, CYBB, NCF1, and TNFSF10 in macrophages. [file Image5.jpeg]

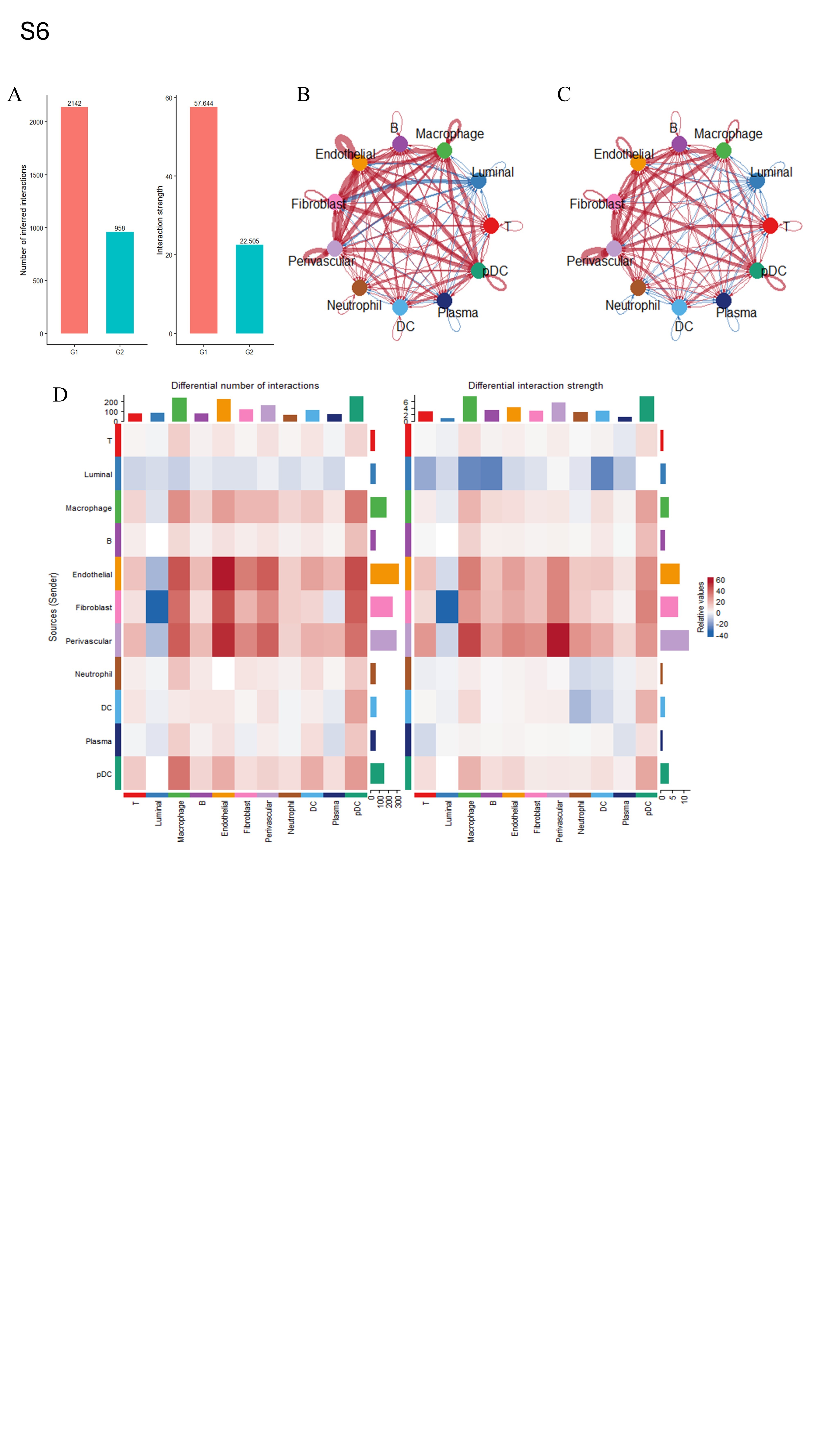

Supplement: Supplementary Figure 6 — Intercellular communication network between GLM and healthy tissues. (A) Quantification of the Number of Inferred Interactions and Interaction Strength; (B) Number of Interactions Between Cell Populations; (C) Interaction Strength Between Cell Populations; (D) Differential Heatmap of Outgoing and Incoming Signaling Across Cell Populations. [file Image6.jpeg]
