# Supplementary material for: Single-cell RNA sequencing analysis reveals cell landscape and gene signatures associated with granulomatous lobular mastitis
Source: Front Immunol. 2025 Oct 16;16:1624640. doi: 10.3389/fimmu.2025.1624640 (PMC12571661; doi:10.3389/fimmu.2025.1624640)
Supplement: Supplementary Table 1 — T cell subtype marker genes. [file Table1.docx]

Supplementary Table S1 T Cell Subtype Marker Genes

| Subtype | Markers |
| --- | --- |
| CD4+ T cell | CD4, IL7R, CCR7, TCF7, LEF1 |
| CD8+ T cell | CD8A, CD8B, GZMB, PRF1, NKG7, CCL5 |
| CD4+ naive T cell | CCR7, SELL, TCF7, IL7R |
| CD4+ memory T cell | S100A4, IL7R, GPR183 |
| CD8+ naive T cell | CCR7, SELL |
| CD8+ effector T cell | GZMB, PRF1, NKG7, CCL5 |
